# Benzothiazole‐endoperoxide conjugates protect PC12 cells against β‐amyloid‐induced cell death via singlet oxygen mediated oxidative detoxification of fibrils

**DOI:** 10.1002/smo.20230019

**Published:** 2024-01-04

**Authors:** Hao Wu, Lei Wang, Xiao Qian, Wanwan Wang, Yu Si, Rensong Sun, Engin U. Akkaya

**Affiliations:** ^1^ State Key Laboratory of Fine Chemicals, and Department of Pharmaceutical Science School of Chemical Engineering Dalian University of Technology Dalian China; ^2^ Global Education Program for AgriScience Frontiers Graduate School of Agriculture Hokkaido University Sapporo Japan

**Keywords:** β‐amyloid protein, Alzheimer's disease, benzothiazole, endoperoxides, singlet oxygen

## Abstract

Metastable endoperoxides with beta‐amyloid fibrils targeting benzothiazole moieties were designed and synthesized. Singlet oxygen released from these endoperoxides by thermal cycloreversion reaction was shown to cause significant structural changes on the amyloid assemblies. Most importantly, the cytotoxicity of the beta‐amyloid fibrils on the PC12 cells were significantly reduced in the presence of endoperoxides. This observation, coupled with the fact that neither external oxygen, nor light is needed for this transformation, is very promising.

## Graphical abstract

Benzothiazole‐endoperoxide conjugates release singlet oxygen by thermal cycloreversion, resulting in a significant increase in the survival rate of PC12 cells by degradation of amyloid fibrils.

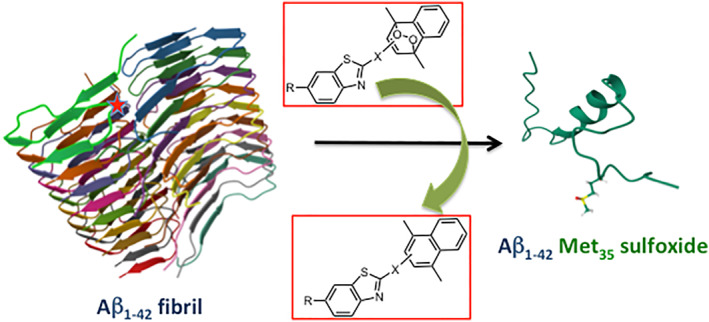

## 1 INTRODUCTION

Alzheimer's disease (AD) is the most common cause of dementia worldwide with an enormous impact on the patients, as well as the families of the patients and the society in general.^[1]^ The main neuropathological indicators of AD are plaques composed of beta‐amyloid protein (Aβ)_1–42_, which is produced by the processing of amyloid precursor protein, and neurofibrillary tangles of highly phosphorylated tau‐proteins.^[2]^ Although challenged by recent clinical studies,[^3^, ^4^, ^5^, ^6^] according to the prevailing idea of the AD pathophysiology known as “the amyloid hypothesis”,^[7]^ abnormal aggregation of Aβ amyloid fibrils is the causative agent of AD, since these fibrils eventually transform into senile plaques, resulting in neurodegeneration and neuronal cell death. There are other ideas suggesting plaques and NFTs to be more of a side product of the actual neurodegenerative transformation, focusing on the involvement of reactive oxygen species (ROS),[^8^, ^9^, ^10^, ^11^, ^12^] metal ions,^[13]^ and insulin resistance.^[14]^ We have previously observed that the term “ROS” may be too broad of a categorization to be able to allow a detailed understanding of diverse actions of these species, especially in the brain.^[15]^

On the other hand, the photodynamic generation of singlet oxygen (^1^O_2_), was shown to degrade Aβ aggregates in vitro, and resulted in the remission of symptoms in disease models developed in C. *elegans*, *Drosophila* and mouse.[^16^, ^17^, ^18^, ^19^, ^20^, ^21^, ^22^, ^23^, ^24^] Experiments with Aβ_1–42_ peptide under the conditions photosensitized generation of singlet oxygen showed that the Met_35_ is a major target for oxidation.[^25^, ^26^] Interestingly, the oxidation of that particular side chain was previously shown to be an important conformational switch which stops the formation of the toxic oligomers and thus, reduces the overall toxicity Aβ.[^27^, ^28^] While photogenerated singlet oxygen does serve as an important mechanistic tool, it does not have practical (clinical) potential, considering the highly impeded tissue penetration of light.[^29^, ^30^, ^31^, ^32^, ^33^, ^34^, ^35^, ^36^] Thus, other means of delivering singlet oxygen to Aβ fibrils are needed. We have shown previously that endoperoxides can replace photogenerated singlet oxygen[^15^, ^37^, ^38^, ^39^, ^40^, ^41^, ^42^] in a number of potential applications, where light penetration is a serious problem.

**FIGURE 1:**
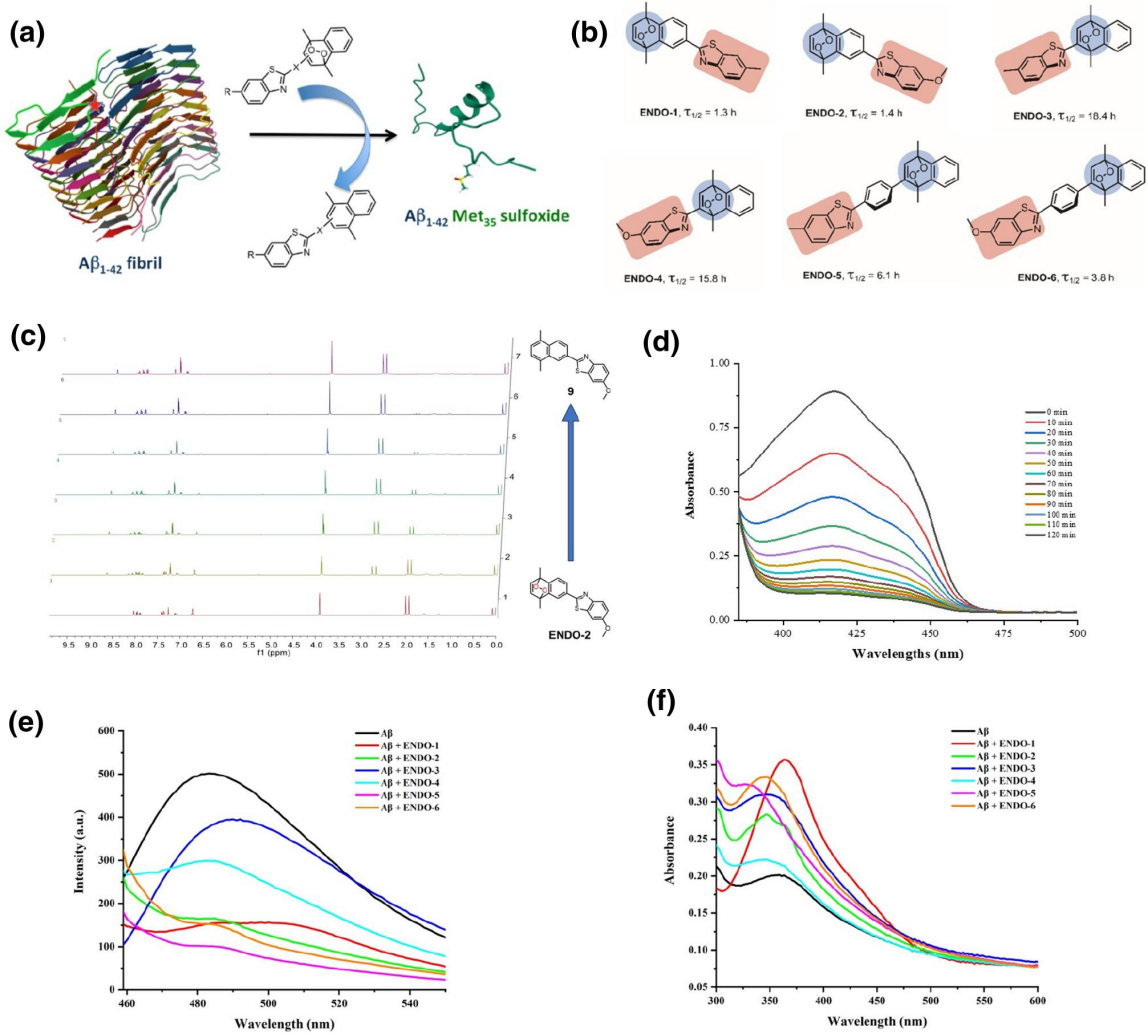
(a) Proposed mode of operation of the BTA‐endoperoxide conjugates targeting Aβ amyloid fibrils. The red star indicates the position of the Met_35_. The solid state nuclear magnetic resonance spectroscopy (NMR) structure of the fibril and the solution NMR structure of the oxidized Aβ_1–42_ Met_35_(SO) were obtained from the Research Collaboratory for Structural Bioinformatics Protein Data Bank (ID numbers 5KK3 and 1BA6, respectively). The red star on the fibril structure indicates the location of Met_35_. (b) benzothiazole (BTA)‐endoperoxide conjugates designed in this work. (c) Temporal evolution of endoperoxide **ENDO‐2** at 37°C. From bottom to top, 1: 0 h, 2: 1 h, 3: 2 h, 4: 3 h, 5: 4 h, 6: 6 h, 7: compound **9**. (d) Time‐dependent UV‐Vis spectra of diphenylisobenzofuran (DPBF) in the presence of 500 μM **ENDO‐2** in dimethylformamide at 37^o^C. (e) Thioflavin T (ThT) emission spectra following typical determination procedure for the 100 μM Aβ, 100 μM Aβ+200 μM various endoperoxide. Excitation at 450 nm. (f) Determination of the absorption spectrum of the 100 μM Aβ and 100 μM Aβ+200 μM various endoperoxide by DNPH method.

β‐Amyloid plaques are known to be selectively stained by Thioflavin T (ThT) fluorescent stain.^[43]^ Over the years, many fluorescent agents have been developed for the fluorescent labeling of amyloid deposits in brain tissue slices.^[44]^ For in vivo imaging, and as an actual diagnostic tool for PET/SPECT techniques, additional factors such as Blood Brain Barrier permeability should be considered.^[45]^ Thus, starting with one of the earliest PET probes for amyloid plaques ^11^C‐PiB,^[46]^ the crucial benzothiazole (BTA) moiety was kept, but the cationic charge was removed. For targeting the endoperoxides to the hydrophobic regions of the Aβ fibrils, we chose to incorporate BTA group into our design (Figure 1a).

## 2 RESULT AND DISCUSSION

With these considerations, we focused on the synthesis of new BTA‐endoperoxide conjugates (Figure 1b) with extended hydrophobic surfaces for targeting hydrophobic regions of oligomeric composed of Aβ_1–42_, and singlet oxygen release potential for oxidative transformation of critical amino acid side chains such as Met_35_. Full details of the synthesis and nuclear magnetic resonance spectroscopy (NMR) characterization for target compounds can be found in Supporting Information (Scheme S1). The rate of singlet oxygen release would be important, as too slow release would be overwhelmed by cellular redox mediators. Too fast release may, in principle, lead to non‐specific reactions and limit conformational switch. The rates of endoperoxide cycloreversion were studied by ^1^H NMR. The changes were indicative of clean cycloreversion reactions, as the spectral data indicates a conversion from endoperoxides to the naphthalene precursors without any by‐products (Figure 1c, Figures S2, S4, S6, S8). Depending on the steric bulk in the vicinity of the endoperoxide bridge, half‐life values spanning a range of 1.3–18.4 h at 37°C were obtained (Figures S1, S3, S5, S7, S9).

**FIGURE 2:**
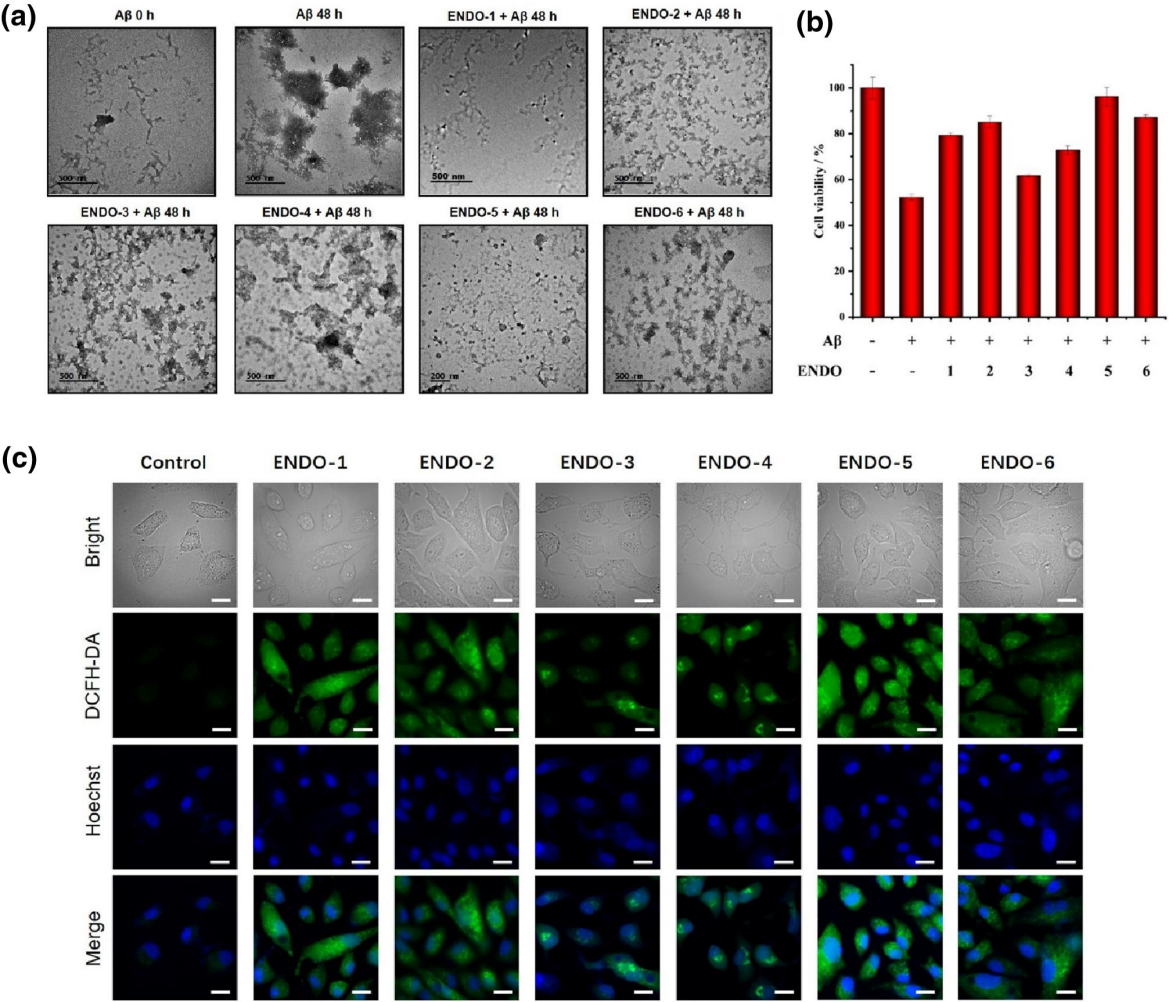
(a) Transmission electron microscopy (TEM) images of 25 μM Aβ monomers, 25 μM Aβ aggregates, and 25 μM Aβ incubated with various endoperoxides (25 μM). (b) Cell viability of PC12 cells as determined by 3‐(4,5‐dimethylthiazol‐2‐yl)‐2,5‐diphenyl‐tetrazolium bromide assays after incubation with or without Aβ protein and **ENDO‐1**, **2**, **3**, **4**, **5** and **6**, respectively. (c) Intracellular imaging of the singlet oxygen release with reactive oxygen species (ROS) probe DCsFH‐DA. PC12 cells incubated with endoperoxides at 20 μM, merged images of DCFH‐DA and Hoechst staining. Scale bar: 20 μm.

Using singlet oxygen trap diphenylisobenzofuran (DPBF), endoperoxides were shown to release singlet oxygen at relative rates commensurate with the half‐lives determined by NMR experiments (Figure 1d, Figures S10‐S13). In order to assess the oxidative degradation activity of endoperoxides via the ThT binding assay, a commonly used strategy for monitoring amyloid fibrils, lyophilized Aβ_1–42_ films were dissolved in ammonium hydroxide solution and then diluted with phosphate buffer. Final solutions contained peptide at 100 μM, final concentration, with or without the endoperoxide compounds (200 μM) and were then incubated at 37°C for 48 h. Following the incubation period, 5 μM of ThT was added and the fluorescence emission data were collected after 5 minutes. As shown in Figure 1e, it clearly shows that all of the endoperoxides seem to limit or counteracted aggregation and/or fibril formation at varying efficiencies. Another method for assessing oxidative transformations of the side chains generating carbonyl groups is the 2,4‐dinitrophenylhydrazine (DNPH) assay (Figure 1f). Aβ_1–42_ oxidized with or without endoperoxides was precipitated with trichloroacetic acid and was further treated with DNPH. As anticipated, increased absorption was found when Aβ_1–42_ was treated with endoperoxides, suggesting that Aβ_1–42_ peptide side chains were efficiently oxidized by singlet oxygen. Transmission electron microscopy (TEM) and dynamic light scattering (DLS) data also provide evidence of the inhibition of the formation of large fibril structures. The TEM picture (Figure 2a) shows that Aβ_1–42_ peptide incubated at 37°C overnight forms large, aggregates of fibrils. However, in the presence of endoperoxides, especially **ENDO‐5**, only very small particles are observed. Similarly, DLS analysis results show that the hydrodynamic volumes of the aggregates are micron‐sized, but they are significantly smaller in the presence of endoperoxides (Figures S14–S20).

For studying the neurotoxicity effects of Aβ‐peptides at cellular level, PC12 cell cultures have proved very useful. PC12 cells are a rat adrenal pheochromocytoma cell line which can differentiate into sympathetic nerve‐like cells by nerve growth factor induction, and are similar to neurons considering physiological/biochemical functions and morphology. Thus, they are considered to be ideal cellular models to study various aspects of AD on the cytotoxicity of Aβ fibrils.^[47]^ First, the toxicity of the endoperoxides and their naphthalene analogs in the range of 0–20 μM were investigated and it was found out that **ENDO‐3** was surprisingly toxic (Figures S21, S22). Then, the cytotoxicity of the Aβ_1–42_, co‐incubated with the endoperoxides was studied with 3‐(4,5‐dimethylthiazol‐2‐yl)‐2,5‐diphenyl‐tetrazolium bromide (MTT) assays (Figure 2b). Obviously, Aβ fibrils are toxic toward PC12 cell and 40 μM of incubated Aβ fibrils lead to 50% decrease in cell viability. Interestingly, all of the endoperoxides provided some protection for the cells, but **ENDO‐5** is remarkable, as cell viability is very close to the control sample. Clearly, the endoperoxide protects PC12 cells against Aβ toxicity. The mechanism is further clarified by imaging intracellular singlet oxygen production. Two‐dye staining of the cells, Hoechst dye for nuclear staining and DCFH‐DA as the ROS probe, and singlet oxygen release was documented (Figure 2c). It is reaffirming to note that slow singlet oxygen releasing endoperoxides **ENDO‐3** and **ENDO‐4** show weaker green emission; as both processes, singlet oxygen release, and the reaction of the available singlet oxygen with the probes are time‐dependent and coupled with other cellular processes.

**FIGURE 3:**
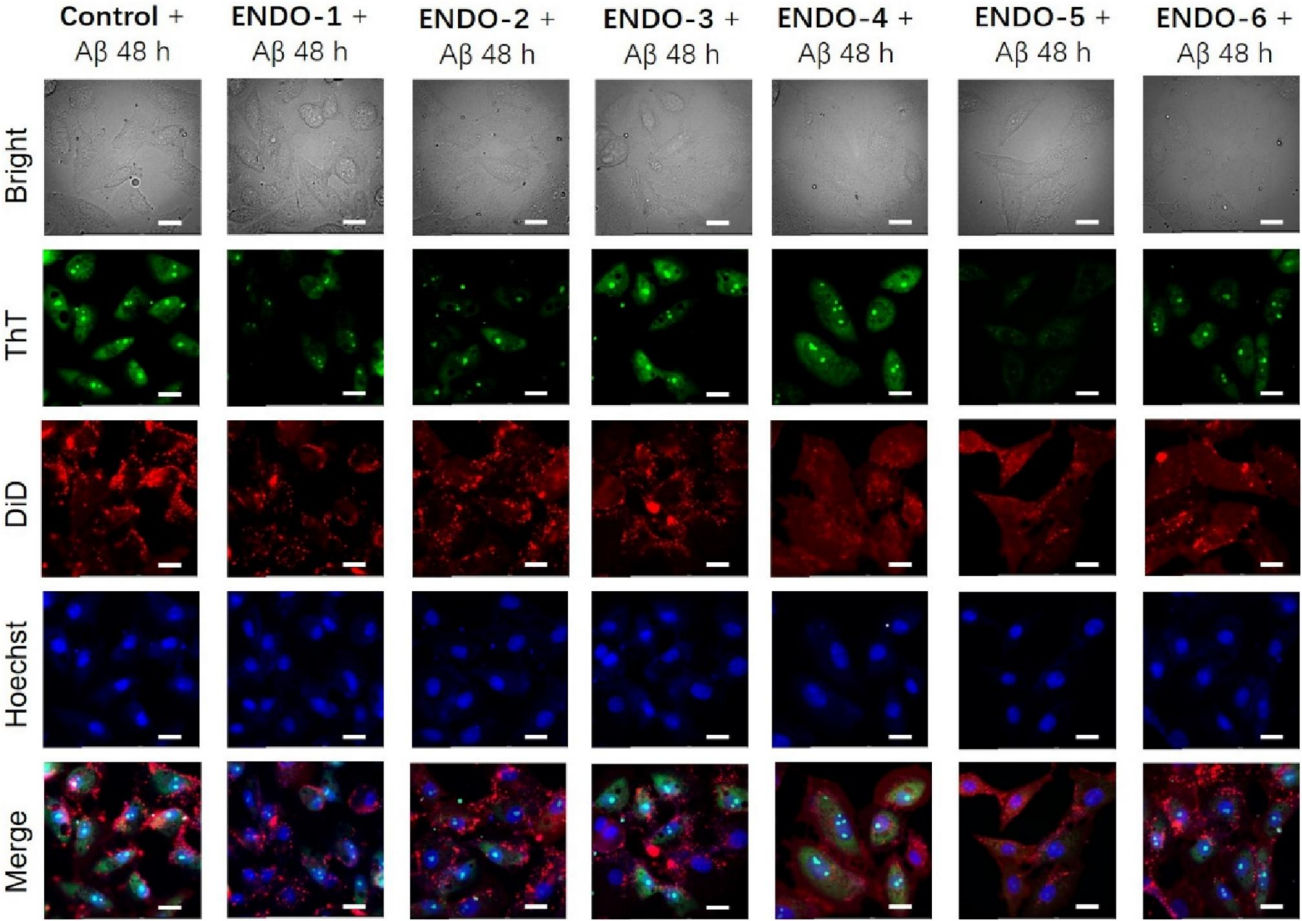
Fluorescence images of PC12 cells treated with Aβ_42_ fibrils, and different endoperoxides mixed with Aβ_42_ fibrils for 48 h, respectively and stained with Thioflavin T (ThT)/DID/Hoechst dyes. For ThT, *λ*_ex_: 405 nm, *λ*_em_: 450–510 nm. For DiD, *λ*_ex_: 635 nm, *λ*_em_: 640–750 nm. For Hoechst, *λ*_ex_: 405 nm, *λ*_em_: 410–450 nm. Scale bar: 20 μm.

The effect of Aβ_1–42_ fibrils on PC12 cells were also investigated microscopically. Thioflavin T dye with a green emission indicates the presence of intact fibril structures. As seen in Figure 3, all endoperoxides decrease the emission intensity to some extent, but **ENDO‐5** is again spectacular as very little green emission is detectable. 1,1′‐dioctadecyl‐3,3,3′,3′‐tetramethylindodicarbocyanine, 4‐chlorobenzenesulfonate (DID) dye is a membrane stain which can provide an understanding of membrane integrity. The merged images also show very clear protective action of **ENDO‐5** for the PC12 cells.

## 3 CONCLUSION

In summary, our results provide strong evidence for the degradation of amyloid fibrils by the BTA‐endoperoxide conjugates with a concomitant increase in the survival rate of PC12 cells. Singlet oxygen release correlates with the observed decrease in Aβ toxicity. Regarding the differences in effectiveness of endoperoxides, a priori prediction of structure‐activity correlation, or even rationalization after experimental work is hampered by the different factors involved. One of them is the hydrophobic surface area exposed to the amyloid structures, but the other factor is the rate of the endoperoxide cycloreversion reaction, which generates singlet oxygen. A larger library of endoperoxide is needed for a thorough understanding and assessment of all the factors involved. It is also highly instructive that one type of ROS, singlet oxygen, is shown to produce a protective effect against amyloid toxicity, especially if targeted properly; whereas another ROS, namely H_2_O_2_, is a well‐known cytotoxic agent for PC12 cells, driving apoptotic cell death at concentrations above 100 μM.^[48]^ Further work along this line is likely to elucidate divergent actions of these two reactive species, and also open a new path toward the reduction of amyloid load in AD patients, with significant potential for therapeutic applications.

## 4 EXPERIMENTAL SECTION/METHODS

Synthetic methods for intermediates are described in Supporting Information.

### 4.1 Synthesis of **ENDO‐2**

Dissolve compound **9** (319.0 mg, 1.0 mmol) in 3.0 mL dichloromethane and cool the mixture to 0°C. A catalytic amount of methylene blue was added and the reaction mixture was stirred under oxygen atmosphere while irradiated with 18 W red light (625 nm). After completion of the reaction, the solvent was removed in vacuum and the crude product was purified by silica gel column chromatography (dichloromethane as eluent) to afford product in 99.7% yield. ^1^H NMR (400 MHz, CDCl_3_) *δ* 8.02 (s, 1H, Ar H), 7.95 (d, *J* = 9.0 Hz, 1H, Ar H), 7.89 (d, *J* = 7.7 Hz, 1H, Ar H), 7.40 (d, *J* = 7.7 Hz, 1H, Ar H), 7.35 (s, 1H, Ar H), 7.10 (d, *J* = 6.4 Hz, 1H, Ar H), 6.75–6.69 (m, 2H, HC=CH), 3.90 (s, 3H, OCH_3_), 1.99 (s, 3H, CH_3_), 1.92 (s, 3H, CH_3_); ^13^C NMR (100 MHz, CDCl_3_) *δ* 165.1 (N=C), 157.9 (Ar C), 148.7 (Ar C), 143.3 (Ar C), 142.0 (Ar C), 139.3 (C=C), 139.0 (C=C), 136.5 (Ar C), 132.4 (Ar C), 126.1 (Ar C), 123.8 (Ar C), 120.7 (Ar C), 118.9 (Ar C), 115.8 (Ar C), 104.2 (Ar C), 78.8 (C=C‐C), 78.5 (C=C‐C), 55.9 (OCH_3_), 16.3 (CH_3_), 16.2 (CH_3_); HRMS‐ESI *m*/*z* calcd for C_20_H_18_NO_3_S [M+H]^+^ 352.1002, found 352.0994.

### 4.2 Synthesis of **ENDO‐3**

Compound **ENDO‐3** was synthesized according to the synthetic method to **ENDO‐2** except that compound **10** was used as the starting material. Yield: 99.8%. ^1^H NMR (400 MHz, CDCl_3_) *δ* 7.82 (d, *J* = 8.3 Hz, 1H, Ar H), 7.58 (s, 1H, Ar H), 7.43–7.38 (m, 1H, Ar H), 7.32–7.28 (m, 1H, Ar H), 7.26–7.19 (m, 3H, Ar H), 7.11 (s, 1H, C=CH), 2.41 (s, 3H, CH_3_), 2.23 (s, 3H, CH_3_), 1.91 (s, 3H, CH_3_); ^13^C NMR (100 MHz, CDCl_3_) *δ* 161.4 (N=C), 152.1 (Ar C), 144.5 (Ar C), 141.14 (Ar C), 141.09 (Ar C), 139.7 (Ar C), 136.0 (C=C), 134.6 (Ar C), 127.9 (Ar C), 127.3 (Ar C), 127.2 (Ar C), 123.1 (Ar C), 121.2 (Ar C), 121.1 (C=C), 120.4 (Ar C), 81.5 (C=C‐C), 78.7 (C=C‐C), 21.6 (CH_3_), 16.3 (CH_3_), 15.7 (CH_3_); HRMS‐ESI *m*/*z* calcd for C_20_H_18_NO_2_S [M+H]^+^ 336.1053, found 336.1043.

### 4.3 Synthesis of **ENDO‐4**

Compound **ENDO‐4** was synthesized according to the synthetic method to **ENDO‐2** except that compound **11** was used as the starting material. Yield: 99.9%. ^1^H NMR (400 MHz, CDCl_3_) *δ* 7.82 (d, *J* = 9.0 Hz, 1H, Ar H), 7.43–7.37 (m, 1H, Ar H), 7.32–7.28 (m, 1H, Ar H), 7.26–7.20 (m, 3H, Ar H), 7.07 (s, 1H, Ar H), 6.99 (s, 1H, C=CH), 3.80 (s, 3H, OCH_3_), 2.23 (s, 3H, CH_3_), 1.91 (s, 3H, CH_3_); ^13^C NMR (100 MHz, CDCl_3_) *δ* 159.9 (N=C), 158.2 (Ar C), 148.6 (Ar C), 144.4 (Ar C), 141.1 (Ar C), 140.7 (Ar C), 139.8 (C=C), 135.9 (Ar C), 127.3 (Ar C), 127.2 (Ar C), 124.2 (Ar C), 121.2 (Ar C), 120.4 (Ar C), 115.8 (C=C), 103.7 (Ar C), 81.5 (C=C‐C), 78.7 (C=C‐C), 55.8 (OCH_3_), 16.3 (CH_3_), 15.7 (CH_3_); HRMS‐ESI *m*/*z* calcd for C_20_H_18_NO_3_S [M+H]^+^ 352.1002, found 352.1094.

### 4.4 Synthesis of **ENDO‐5**

Compound **ENDO‐5** was synthesized according to the synthetic method to **ENDO‐2** except that compound **12** was used as the starting material. Yield: 99.6%. ^1^H NMR (400 MHz, CDCl_3_) *δ* 7.98 (d, *J* = 8.2 Hz, 2H, Ar H), 7.88 (d, *J* = 8.3 Hz, 1H, Ar H), 7.63 (s, 1H, Ar H), 7.33–7.30 (m, 2H, Ar H), 7.27–7.23 (m, 5H, Ar H), 6.54 (s, 1H, C=CH), 2.43 (s, 3H, CH_3_), 1.88 (s, 3H, CH_3_), 1.73 (s, 3H, CH_3_); ^13^C NMR (100 MHz, CDCl_3_) *δ* 165.3 (N=C), 151.2 (Ar C), 149.8 (Ar C), 140.2 (C=C), 140.0 (Ar C), 137.6 (Ar C), 134.8 (Ar C), 134.6 (Ar C), 134.2 (Ar C), 132.3 (Ar C), 127.8 (Ar C), 127.0 (Ar C), 126.2 (Ar C), 126.0 (Ar C), 125.9 (Ar C), 121.7 (Ar C), 120.4 (Ar C), 119.3 (C=C), 119.2 (Ar C), 80.2 (C=C‐C), 77.9 (C=C‐C), 20.6 (CH_3_), 15.3 (CH_3_), 14.3 (CH_3_); HRMS‐ESI *m*/*z* calcd for C_26_H_22_NO_2_S [M+H]^+^ 412.1366, found 412.1354.

### 4.5 Synthesis of **ENDO‐6**

Compound **ENDO‐6** was synthesized according to the synthetic method to **ENDO‐2** except that compound **13** was used as the starting material. Yield: 99.8%. ^1^H NMR (400 MHz, CDCl_3_) *δ* 7.95 (d, *J* = 8.2 Hz, 2H, Ar H), 7.88 (d, *J* = 8.9 Hz, 1H, Ar H), 7.33–7.29 (m, 3H, Ar H), 7.27–7.23 (m, 4H, Ar H), 7.03 (d, *J* = 9.0 Hz, 1H, Ar H), 6.54 (s, 1H, C=CH), 3.83 (s, 3H, OCH_3_), 1.88 (s, 3H, CH_3_), 1.73 (s, 3H, CH_3_); ^13^C NMR (100 MHz, CDCl_3_) *δ* 164.8 (N=C), 157.9 (Ar C), 150.9 (Ar C), 148.7 (C=C), 141.3 (Ar C), 141.1 (Ar C), 138.4 (Ar C), 136.5 (Ar C), 135.8 (Ar C), 133.4 (Ar C), 128.8 (Ar C), 127.1 (Ar C), 127.0 (Ar C), 123.8 (Ar C), 120.3 (Ar C), 120.2 (Ar C), 115.8 (C=C), 104.2 (Ar C), 81.3 (C=C‐C), 78.9 (C=C‐C), 55.9 (OCH_3_), 16.3 (CH_3_), 15.3 (CH_3_); HRMS‐ESI *m*/*z* calcd for C_26_H_22_NO_3_S [M+H]^+^ 428.1315, found 428.1307.

### 4.6 Temporal evolution of endoperoxides

Temporal evolution of endoperoxides was performed by ^1^H NMR and it was observed that the **ENDO‐2**, **3**, **4**, **5** and **6** showed half‐life of 1.4 h, 18.4 h, 15.8 h, 6.1 and 3.8 h at 37°C, respectively. The rate constant and half‐life calculations were calculated using first‐order reaction rate equations. The equations are as follows: ln[*A*] = ‐*kt* + ln[*A*]_0_, *t*_1/2_ = 0.693/*k*.

### 4.7 Singlet oxygen detection

A singlet oxygen probe, 1,3‐diphenylisobenzofuran (DPBF), was utilized to detect singlet oxygen release from endoperoxide. In brief, 500 μM endoperoxide was combined with 50 μM DPBF in dimethylformamide, and the UV‐Vis measurements were taken at 37°C in the dark. The release of singlet oxygen from endoperoxide was detected by a decrease in absorbance at 417 nm.

### 4.8 Monomeric Aβ_1–42_ solution preparation

In order to obtain film‐like lyophilized Aβ_1–42_, Aβ_1–42_ (5 mg) was first dissolved in hexafluoro‐2‐propanol (HFIP) and the solution was subsequently divided into microcentrifuge tubes (0.2 mg aliquots), then placed at room temperature overnight, and finally placed in a vacuum desiccator for drying process. The obtained monomeric Aβ_1–42_ films were kept at −20°C and used in subsequent studies.

### 4.9 Thioflavin T assay

The Aβ_1–42_ film was re‐dissolved in ammonium hydroxide (20 μL) and subsequently diluted to 100 μM using 10 mM phosphate buffer (pH 7.4).The peptide (20 μL, 100 μM, final concentration) was then incubated with or without endoperoxide (200 μM, final concentration) for 48 h at 37°C. The sample was diluted to a final volume of 200 μL with 50 mM glycine‐NaOH buffer (pH 8.0) containing ThT (5 μM) after incubation. After 5 min, the fluorescence intensities were measured (excitation 450 nm).

### 4.10 DNPH assay

Aβ_1–42_ peptide (20 μL, 100 μM, final concentration) was incubated with or without endoperoxides (20 μL, 200 μM, final concentration) for 48 h at 37°C. The peptide Aβ_1–42_ was then incubated in an ice bath with 500 μL of trichloroacetic acid (TCA, 20% final concentration). The Aβ_1–42_ protein was then treated with 500 μL of trichloroacetic acid (TCA, 20% final concentration) solution in an ice bath for 15 min, followed by recovery by centrifugation (5 min, 14,000 rpm). Next, 400 μL of 10 mM DNPH in 2 M HCl solution was added to each tube, followed by incubation for 1 h at room temperature. Each sample was then precipitated with 20% TCA solution and washed with 600 μL ethanol‐ethyl acetate (1:1, v/v) solution. The samples in guanosine hydrochloride solution (600 μL, 6 M, pH 2.3) were reintroduced at 37°C for 20 min, after which the privatization was determined using UV‐vis spectroscopy.

### 4.11 Transmission electron microscopy analysis

To inhibit Aβ1–42 aggregation, monomeric Aβ_1–42_ was treated with different endoperoxides, respectively, for 48 h at 37°C. In addition, monomeric Aβ_1–42_ and aggregated Aβ_1–42_ were used as controls. The experimental concentrations of Aβ_1–42_ and endoperoxides were set at 25 μM (final concentration). An aliquot (10 μL) of the sample was deposited on a carbon‐coated grid of Cu/Rh for 2 min at room temperature. Each grid was then stained with a 0.5% phosphotungstate (PTA) solution. After the removal of excess staining solution, the samples were transferred for imaging using a TEM (JEM‐1400Flash).

### 4.12 Dynamic light scattering measurement

To inhibit Aβ_1–42_ aggregation, monomeric Aβ_1–42_ was treated with different endoperoxides, respectively, for 48 h at 37°C. Moreover, monomeric Aβ_1–42_ and aggregated Aβ_1–42_ were used as controls. The experimental concentrations of Aβ_1–42_ and endoperoxides were set at 25 μM (final concentration). Equivalent samples (100 μL) were subsequently diluted to 2 mL using phosphate buffered saline (PBS). finally, each sample was analyzed using a Zetapotential analyzer to obtain DLS (ZS90).

### 4.13 Inhibition of Aβ_1–42_ aggregation in vitro

PC12 cells were seeded in 96‐well plates (5000 cells per well) and treated in dulbecco's modified eagle medium (DMEM) medium for 24 h at 37°C. Then, the cell was co‐incubated for another 48 h at 37°C with monomeric Aβ_1–42_ (40 μM) or a mixture of monomeric Aβ_1–42_ (40 μM) and endoperoxides (20 μM), respectively. Following that, 20 μL 5 mg/mL MTT was added to each well. The media was removed after a further 4 h of incubation at 37°C. Dimethylsulfoxide (150 μL) was added to each well and the absorbance at 570 nm was measured using a microplate reader (SpectraMax i3x Molecular Devices).

### 4.14 Intracellular singlet oxygen generation

PC12 cells (1.5 × 10^5^ cells per dish) were seeded and cultured for 24 h to allow proper adhesion of the cells to the glass bottom dishes. Subsequently, the endoperoxides (20 μM) were added to different dishes for incubation for 6 h, respectively. Following this, the medium was replaced and washed with PBS. The cells were incubated for 45 min at 37°C after being treated with DCFH‐DA (10 M) in DMEM. The solution was removed after the incubation, and the cells were washed with PBS. Cell nuclei were stained with Hoechst for 15 min. The extra Hoechst was washed with PBS and the imaging was observed with a Fluorescence microscope (DMi8).

### 4.15 Fluorescence imaging of Aβ_1–42_ fibrils

PC12 cells were seeded and cultured in a dish at 1.5 × 10^5^ cells/dish for 24 h. Then, monomeric Aβ_1–42_ (40 μM) and the mixture of monomeric Aβ_1–42_ (40 μM) and endoperoxides (20 μM) were respectively added to different dishes for incubation for 48 h. After that, the medium was replaced and washed with PBS three times. The cells were incubated with ThT (30 μM, 1 mL) for 30 min. Cell membranes and nuclei were respectively stained with DiD and Hoechst for 10 min and 15 min. The fluorescence images were obtained by Fluorescence microscope (DMi8).

## CONFLICT OF INTEREST STATEMENT

The authors declare no conﬂicts of interest.

## Supporting information

Supplementary Material

## Data Availability

Data available in article supplementary material.

## References

[1] A. Burns , S. Iliffe , BMJ 2009, 338, 405.

[2] M. A. DeTure , D. W. Dickson , Mol. Neurodegener. 2019, 14, 32. 31375134 10.1186/s13024-019-0333-5 PMC6679484

[3] E. Karran , B. De Strooper , Nat. Rev. Drug Discov. 2021, 21, 306. 10.1038/s41573-022-00391-w 35177833

[4] D. J. Selkoe , Neuron 1991, 6, 487. 1673054 10.1016/0896-6273(91)90052-2

[5] J. A. Hardy , G. A. Higgins , Science 1992, 256, 184. 1566067 10.1126/science.1566067

[6] I. W. Hamley , Chem. Rev. 2012, 112, 5147. 22813427 10.1021/cr3000994

[7] D. R. Thal , M. Fändrich , Acta Neuropathol. 2015, 129, 163. 25600324 10.1007/s00401-015-1387-2

[8] M. A. Ansari , S. W. Scheff , J. Neuropathol. Exp. Neurol. 2010, 69, 155. 20084018 10.1097/NEN.0b013e3181cb5af4 PMC2826839

[9] P. Youssef , B. Chami , J. Lim , T. Middleton , G. Sutherland , P. Witting , Sci. Rep. 2018, 8, 11553. 30068908 10.1038/s41598-018-29770-3 PMC6070512

[10] D. Boyd‐Kimball , R. Sultana , H. Fai Poon , B. C. Lynn , F. Casamenti , G. Pepeu , J. B. Klein , D. A. Butterfield , Neurosci 2005, 132, 313. 10.1016/j.neuroscience.2004.12.022 15802185

[11] M. Arimon , S. Takeda , K. Post , S. Svirsky , B. Hyman , O. Berezovska , Neurobiol. Dis. 2015, 84, 109. 26102023 10.1016/j.nbd.2015.06.013 PMC4684986

[12] D. Praticò , K. Uryu , S. Leight , J. Trojanoswki , V. Lee , J. Neurosci. 2001, 21, 4183. 11404403 10.1523/JNEUROSCI.21-12-04183.2001 PMC6762743

[13] Y. Liu , M. Nguyen , A. Robert , B. Meunier , Acc. Chem. Res. 2019, 52, 2026. 31274278 10.1021/acs.accounts.9b00248

[14] D. Bosco , A. Fava , M. Plastino , T. Montalcini , A. Pujia , J. Cell Mol. Med. 2011, 15, 1807. 21435176 10.1111/j.1582-4934.2011.01318.x PMC3918038

[15] H. Wu , Z. Liu , Y. Shao , G. Li , Y. Pan , L. Wang , E. Akkaya , Chem. Commun. 2022, 58, 3747. 10.1039/d1cc07133e 35072189

[16] H. Yagi , D. Ozawa , K. Sakurai , T. Kawakami , H. Kuyama , O. Nishimura , T. Shimanouchi , R. Kuboi , H. Naiki , Y. Goto , J. Biol. Chem. 2010, 285, 19660. 20406822 10.1074/jbc.M109.076505 PMC2885244

[17] A. Taniguchi , Y. Shimizu , K. Oisaki , Y. Sohma , M. Kanai , Nat. Chem. 2016, 8, 974. 27657874 10.1038/nchem.2550

[18] A. Taniguchi , D. Sasaki , A. Shiohara , T. Iwatsubo , T. Tomita , Y. Sohma , M. Kanai , Angew. Chem. Int. Ed. 2014, 53, 1382. 10.1002/anie.201308001 24339209

[19] J. Lee , B. Lee , C. Park , Biomaterials 2015, 38, 43. 25457982 10.1016/j.biomaterials.2014.10.058

[20] A. Hirabayashi , Y. Shindo , K. Oka , D. Takahashi , K. Toshima , Chem. Commun. 2014, 50, 9543. 10.1039/c4cc03791j 25012260

[21] B. Lee , S. Lee , Y. Suh , J. Lee , A. Kim , O. Kwon , K. Yu , C. Park , Angew. Chem. Int. Ed. 2015, 54, 11472. 10.1002/anie.201504310 26178411

[22] B. I. Lee , Y. S. Suh , Y. Chung , K. Yu , C. Park , Sci. Rep. 2017, 7, 7523. 28790398 10.1038/s41598-017-07581-2 PMC5548810

[23] Y. J. Hsieh , K. Y. Chien , I. Yang , I. Lee , C. Wu , T. Huang , J. Yu , Sci. Rep. 2017, 7, 1370. 28465586 10.1038/s41598-017-01409-9 PMC5431048

[24] M. W. Beck , J. S. Derrick , R. Kerr , S. Oh , W. Cho , S. Lee , Y. Ji , J. Han , Z. Tehrani , N. Suh , S. Kim , S. Larsen , K. Kim , J. Lee , B. Ruotolo , M. Lim , Nat. Commun. 2016, 7, 13115. 27734843 10.1038/ncomms13115 PMC5065625

[25] M. Friedemann , E. Helk , A. Tiiman , K. Zovo , P. Palumaa , V. Tougu , Biochem. Biophys. Rep. 2015, 3, 94. 29124171 10.1016/j.bbrep.2015.07.017 PMC5668694

[26] F. Maghsoodi , T. D. Martin , E. Chi , ACS Omega 2023, 8, 10148. 36969430 10.1021/acsomega.2c07468 PMC10035002

[27] L. Hou , H. Shao , Y. Zhang , H. Li , N. Menon , E. Neuhaus , J. Brewer , I. Byeon , D. Ray , M. Vitek , T. Iwashita , R. Makula , A. Przybyla , M. Zagorski , J. Am. Chem. Soc. 2004, 126, 1992. 14971932 10.1021/ja036813f

[28] G. Bitan , B. Tarus , S. Vollers , H. Lashuel , M. Condron , J. Straub , D. Teplow , J. Am. Chem. Soc. 2003, 125, 15359. 14664580 10.1021/ja0349296

[29] S. Stolik , J. Delgado , A. Perez , L. Anasagasti , J. Photochem. Photobiol., B 2000, 57, 90. 11154088 10.1016/s1011-1344(00)00082-8

[30] P. Lapchak , P. Boitano , P. Butte , D. Fisher , T. Holscher , E. Ley , M. Nuno , A. Voie , P. Rajput , PLoS One 2015, 10, e0127580. 26039354 10.1371/journal.pone.0127580 PMC4454538

[31] F. Salehpour , P. Cassano , N. Rouhi , M. Hamblin , L. Taboada , F. Farajdokht , J. Mahmoudi , Photomed. Photobiomodul. Laser Surg. 2019, 37, 581. 10.1089/photob.2019.4676 31553265

[32] H. Eggert , V. Blazek , Neurosurg 1987, 21, 459. 10.1227/00006123-198710000-00003 3683777

[33] M.‐R. Kim , S. S. Park , J. Han , T. C. Pham , M. Kang , S. Lee , Bull. Korean Chem. Soc. 2023, 1. 10.1002/bkcs.12781

[34] M.‐R. Kim , T. C. Pham , H.‐S. Yang , S. H. Park , S. Lee , Bull. Korean Chem. Soc. 2022, 43, 1141.

[35] H. Kim , Y. R. Lee , H. Jeong , J. Lee , X. Wu , H. Li , J. Yoon , Smart Mol. 2023, 1, e20220010. 10.1002/smo.20220010 PMC12118215 40625653

[36] H. Kim , M. Yang , N. Kwon , M. Cho , J. Han , R. Wang , S. Qi , H. Li , V.‐N. Nguyen , X. Li , H.‐B. Cheng , J. Yoon , Bull. Korean Chem. Soc. 2023, 44, 236.

[37] H. Wu , L. Wang , Y. Wang , Y. Shao , G. Li , K. Shao , E. U. Akkaya , Angew. Chem. Int. Ed. 2022, 61, e202210249. 10.1002/anie.202210249 36082673

[38] L. Wang , L. Tang , Y. Liu , H. Wu , Z. Liu , J. Li , Y. Pan , E. U. Akkaya , Chem. Commun. 2022, 58, 1902. 10.1039/d1cc05810j 35029263

[39] M. Liu , E. Ucar , Z. Liu , L. Wang , L. Yang , J. Xu , E. U. Akkaya , RSC Adv. 2021, 11, 14513. 35423982 10.1039/d1ra02569d PMC8697772

[40] M. Qu , N. Wu , W. Jiang , L. Wang , M. Akkaya , E. U. Akkaya , RSC Adv. 2021, 11, 19083. 35478644 10.1039/d1ra02933a PMC9033937

[41] S. Ayan , G. Gunaydin , N. Yesilgul‐Mehmetcik , M. Gedik , O. Seven , E. U. Akkaya , Chem. Commun. 2020, 56, 14793. 10.1039/d0cc06031c 33196713

[42] E. Ucar , D. Xi , O. Seven , C. Kaya , X. Peng , W. Sun , E. U. Akkaya , Chem. Commun. 2019, 55, 13808. 10.1039/c9cc06231a 31613284

[43] H. Naiki , K. Higuchi , M. Hosokawa , T. Takeda , Anal. Biochem. 1989, 177, 244. 2729542 10.1016/0003-2697(89)90046-8

[44] S. Jung , S. Park , E. Lee , J. Park , Y. Kong , J. Rho , M. Hur , S. Yang , Y. Park , Arch Pharm. Res. (Seoul) 2015, 38, 1992. 10.1007/s12272-015-0617-4 26012373

[45] P. Ballabh , A. Braun , M. Nedergaard , Neurobiol. Dis. 2004, 16, 1. 15207256 10.1016/j.nbd.2003.12.016

[46] W. Klunk , H. Engler , A. Nordberg , Y. Wang , G. Blomqvist , D. Holt , M. Bergstrom , I. Savitcheva , G. Huang , S. Estrada , B. Ausen , M. Debnath , J. Barletta , J. Price , J. Sandell , B. Lopresti , A. Wall , P. Koivisto , G. Antoni , C. Mathis , B. Langstrom , Ann. Neurol. 2004, 55, 306. 14991808 10.1002/ana.20009

[47] D. Xie , T. Deng , Z. Zhai , T. Sun , Y. Xu , Front. Mol. Neurosci. 2003, 15, 1016559. 10.3389/fnmol.2022.1016559 PMC9846650 36683856

[48] X. Lin , S. Wu , Y. Shi , G. Liu , J. Zhi , F. Wang , Cell. Mol. Neurobiol. 2016, 36, 1365. 26961382 10.1007/s10571-016-0336-5 PMC11482298

