# Supplementary material for: Benzothiazole‐endoperoxide conjugates protect PC12 cells against β‐amyloid‐induced cell death via singlet oxygen mediated oxidative detoxification of fibrils
Source: Smart Mol. 2024 Jan 4;2(2):e20230019. doi: 10.1002/smo.20230019 (PMC12118204; doi:10.1002/smo.20230019)
Supplement: Supplementary file 1 — Supplementary Material [file SMO2-2-e20230019-s001.docx]

Supporting Information

**Benzothiazole-endoperoxide conjugates protect PC12 cells against β-amyloid-induced cell death via singlet oxygen mediated oxidative detoxification of fibrils**

Hao Wu, Lei Wang,* Xiao Qian, Wanwan Wang, Yu Si, Rensong Sun, and Engin U. Akkaya*

**Table of Contents**

[1. General Experimental Information 3](#_Toc148013659)

[2. Synthesis of Intermediates and Endoperoxides 4](#_Toc148013660)

[3. Temporal Evolution of Endoperoxides 9](#_Toc148013661)

[4. Detection of Singlet Oxygen 13](#_Toc148013662)

[5. Dynamic Light Scattering (DLS) Measurement 15](#_Toc148013663)

[6. Cytotoxicity of Endoperoxides 18](#_Toc148013664)

[7. Reference 19](#_Toc148013665)

[8. NMR Spectra 20](#_Toc148013666)

# 1. General Experimental Information

**Materials:** Unless otherwise specified, all reagents and solvents were purchased from commercial suppliers and used without further purification. Reactions were monitored by thin layer chromatography using Huang-hai TLC Silica gel 60 F-254. Column chromatography was performed by using Mei-gao Silica Gel 60 (particle size: 200-300 mesh). 1,3-Diphenylisobenzofuran (DPBF), 2,4-Dinitrophenylhydrazine (DNPH) and Thioflavin T (ThT) were purchased from Shanghai aladdin Co., Ltd (Shanghai, China). Reactive Oxygen Species Assay Kit and Hoechst staining solution were bought from Beijing Solarbio Science & Technology Co., Ltd (Beijing, China). DiD Kit were bought from Shanghai Beyotime Biotechnology Co., Ltd (Shanghai, China).

**Instruments:** The ^1^H and ^13^C NMR spectra were recorded using Bruker Vaian DLG400. Chemical shifts were reported in parts per million (ppm) and coupling constants (*J* values) are given in Hz. Splitting patterns are indicated as follow s, singlet; d, doublet; t, triplet; m, multiplet. Mass spectra were recorded with Q Exactive Plus (Thermo Fisher, USA). The UV-Vis absorption spectra were performed by using Agilent Cary-3500 UV-Vis spectrophotometer. Fluorescence spectra were determined on Agilent CARY Eclipse spectrophotometer. The MTT assay was performed on a SpectraMax i3x microplate reader (Molecular Devices, USA). TEM imaging were determined on JEM-1400 Flash. Dynamic Light Scattering (DLS) measurement was performed by using Zetapotential analyzer (ZS90). Fluorescence imaging were determined on Fluorescence microscope DMi8.

# 2. Synthesis of Intermediates and Endoperoxides

**Scheme S1**. The syntheses of endoperoxides.

Compound **2**-**4, 8** and **ENDO-1** were synthesized following our previous work.^[1]^

*Synthesis of* ***5***^[2]^: 1,4-Dimethylnaphthalene **1** (1.56 g, 10.0 mmol) was dissolved in 15 mL chloroform under argon atmosphere and exclusion of light. Bromine solution (0.54 mL, 10.5 mmol) was added to the reaction mixture over 10 minutes at 0 °C and the reaction mixture was stirred at room temperature for 2 hours. Then, the reaction mixture was diluted with 20 mL chloroform and washed with 25 mL saturated Na_2_S_2_O_3_ solution, 25 mL water, and 25 mL saturated NaCl solution, respectively. Organic layer was combined and dried over anhydrous Na_2_SO_4_. The solution was filtered through a thin pad of silica gel. After removal of the solvent by rotary evaporator, the crude product was purified by silica gel column chromatography using hexane as the eluent. The product was obtained in colorless oil form in 93% yield. ^1^H NMR (400 MHz, CDCl_3_) *δ* 8.05 − 8.01 (m, 1H, Ar H), 7.97 − 7.93 (m, 1H, Ar H), 7.55 − 7.51 (m, 2H, Ar H), 7.47 (s, 1H, Ar H), 2.76 (s, 3H, CH_3_), 2.62 (s, 3H, CH_3_).

*Synthesis of* ***6*:** To a solution of the compound **5** (117.5 mg, 0.5 mmol) in anhydrous Et_2_O (4 mL), *n*-BuLi (1.6 M in *n*-hexane, 0.31 mL, 0.5 mmol) was added dropwise. After stirring for 1 h, anhydrous DMF (0.05 mL) was added under vigorous stirring and the mixture was stirred for 2 h. A saturated NH_4_Cl aq solution was added to the reaction mixture and the mixture was extracted with Et_2_O. The ethereal phase was washed with H_2_O, dried (Na_2_SO_4_) and concentrated in vacuo. The crude product was purified by column chromatography using Hexane: EtOAc (20:1, v/v) in 50% yield. ^1^H NMR (400 MHz, CDCl_3_) *δ* 10.56 (s, 1H, CHO), 8.17 (d, *J* = 8.2 Hz, 1H, Ar H), 7.95 (d, *J* = 9.1 Hz, 1H, Ar H), 7.67 (s, 1H, Ar H), 7.61 – 7.51 (m, 2H, Ar H), 2.92 (s, 3H, CH_3_), 2.61 (s, 3H, CH_3_); ^13^C NMR (100 MHz, CDCl_3_) *δ* 191.1 (C=O), 137.4 (Ar C), 134.3 (Ar C), 132.0 (Ar C), 131.9 (Ar C), 129.7 (Ar C), 127.5 (Ar C), 125.4 (Ar C), 124.7 (Ar C), 123.8 (Ar C), 123.4 (Ar C), 18.3 (CH_3_), 11.7 (CH_3_).

*Synthesis of* ***7*:** Potassium carbonate (138 mg, 1 mmol), compound **5** (117.5 mg, 0.5 mmol), THF (2 mL), and water (1 mL) were mixed and stirred at room temperature. After purged with argon, Pd(Ph_3_P)_4_ (25 mg, 0.025 mmol), *p*-boronobenzaldehyde (74.7 mg, 0.5 mmol) were added into the reaction mixture, which was then refluxed under Ar atmosphere. After completion, the mixture was cooled to room temperature, extracted with EtOAc (3 × 20 mL), and the organic layer was washed with water (3 × 20 mL), saturated NaCl solution (3 × 20 mL) and dried over anhydrous Na_2_SO_4_. After removal of the solvent in vacuo, the crude product was purified by silica gel column chromatography with Hexane: EtOAc (100:1, v/v) to afford product in 64% yield. ^1^H NMR (400 MHz, CDCl_3_) *δ* 10.12 (s, 1H, CHO), 8.17 (d, *J* = 9.7 Hz, 1H, Ar H), 8.10 (d, *J* = 9.6 Hz, 1H, Ar H), 8.00 (d, *J* = 8.1 Hz, 2H, Ar H), 7.67 – 7.60 (m, 2H, Ar H), 7.59 (d, *J* = 8.1 Hz, 2H, Ar H), 7.27 (s, 1H, Ar H), 2.75 (s, 3H, CH_3_), 2.62 (s, 3H, CH_3_); ^13^C NMR (100 MHz, CDCl_3_) *δ* 191.0 (C=O), 148.3 (Ar C), 136.3 (Ar C), 133.8 (Ar C), 132.0 (Ar C), 131.2 (Ar C), 129.5 (Ar C), 128.5 (Ar C), 128.0 (Ar C), 127.2 (Ar C), 125.1 (Ar C), 124.8 (Ar C), 124.1 (Ar C), 123.6 (Ar C), 18.2 (CH_3_), 15.1 (CH_3_).

*Synthesis of* ***9*:** YCl_3_ (9.8 mg, 0.05 mmol) were dissolved in 10 mL EtOH and stirred until the solid dissolved completely under refluxing, then, 2-amino-5-methoxybenzenethiol (170 mg, 1.1 mmol) and compound **4** (184 mg, 1.0 mmol) was added into the reaction mixture. After 5 hours, the reaction mixture was cooled to room temperature, and evaporated under vacuum. The product was purified by column chromatography with Hexane: EtOAc (50:1, v/v) to afford product in 62% yield.^1^H NMR (400 MHz, CDCl_3_) *δ* 8.67 (s, 1H, Ar H), 8.19 (d, *J* = 7.0 Hz, 1H, Ar H), 8.09 (d, *J* = 8.8 Hz, 1H, Ar H), 8.00 (d, *J* = 8.9 Hz, 1H, Ar H), 7.39 (s, 1H, Ar H), 7.27 (s, 2H, Ar H), 7.12 (d, *J* = 8.9 Hz, 1H, Ar H), 3.92 (s, 3H, OCH_3_), 2.78 (s, 3H, CH_3_), 2.69 (s, 3H, CH_3_); ^13^C NMR (100 MHz, CDCl_3_) *δ* 166.0 (N=C), 157.8 (Ar C), 148.9 (Ar C), 136.6 (Ar C), 133.8 (Ar C), 133.3 (Ar C), 132.7 (Ar C), 132.5 (Ar C), 130.5 (Ar C), 127.7 (Ar C), 127.3 (Ar C), 125.6 (Ar C), 124.0 (Ar C), 123.8 (Ar C), 123.7 (Ar C), 115.6 (Ar C), 104.2 (Ar C), 55.8 (OCH_3_), 19.5 (CH_3_), 19.3 (CH_3_); HRMS-ESI *m*/*z* calcd for C_20_H_18_NOS [M+H]^+^ 320.1104, found 320.1100.

*Synthesis of* ***10*:** Compound **10** was synthesized according to the synthetic method to compound **9** except that compound **6** and 2-amino-5-methylbenzenethiol were used instead of compound **4** and 2-amino-5-methoxybenzenethiol, respectively. Yield: 85%. ^1^H NMR (400 MHz, CDCl_3_) *δ* 8.15 – 8.12 (m, 1H, Ar H), 7.99 – 7.97 (m, 1H, Ar H), 7.95 (d, *J* = 8.3 Hz, 1H, Ar H), 7.67 (s, 1H, Ar H), 7.55 – 7.52 (m, 3H, Ar H), 7.27 (d, *J* = 8.3 Hz, 1H, Ar H), 2.84 (s, 3H, CH_3_), 2.65 (s, 3H, CH_3_), 2.46 (s, 3H, CH_3_); ^13^C NMR (100 MHz, CDCl_3_) *δ* 166.8 (N=C), 150.8 (Ar C), 135.3 (Ar C), 134.2 (Ar C), 132.15 (Ar C), 132.1 (Ar C), 131.6 (Ar C), 131.0 (Ar C), 129.1 (Ar C), 126.9 (Ar C), 126.7 (Ar C), 125.5 (Ar C), 125.2 (Ar C), 124.7 (Ar C), 123.6 (Ar C), 121.9 (Ar C), 120.1 (Ar C), 20.6 (CH_3_), 18.3 (CH_3_), 15.1 (CH_3_); HRMS-ESI *m*/*z* calcd for C_20_H_18_NS [M+H]^+^ 304.1154, found 304.1151.

*Synthesis of* ***11*:** Compound **11** was synthesized according to the synthetic method to compound **9** except that compound **6** was used instead of compound **4**. Yield: 25%. ^1^H NMR (400 MHz, CDCl_3_) *δ* 8.15 – 8.11 (m, 1H, Ar H), 8.00 – 7.93 (m, 2H, Ar H), 7.56 – 7.50 (m, 3H, Ar H), 7.33 (d, *J* = 2.5 Hz, 1H, Ar H), 7.06 (d, *J* = 8.9 Hz, 1H, Ar H), 3.85 (s, 3H, OCH_3_), 2.84 (s, 3H, CH_3_), 2.65 (s, 3H, CH_3_); ^13^C NMR (100 MHz, CDCl_3_) *δ* 165.3 (N=C), 156.7 (Ar C), 147.3 (Ar C), 136.5 (Ar C), 132.2 (Ar C), 132.1 (Ar C), 131.6 (Ar C), 131.0 (Ar C), 129.0 (Ar C), 126.9 (Ar C), 125.5 (Ar C), 125.2 (Ar C), 124.7 (Ar C), 123.6 (Ar C), 122.9 (Ar C), 114.5 (Ar C), 102.9 (Ar C), 54.8 (OCH_3_), 18.3 (CH_3_), 15.0 (CH_3_). HRMS-ESI *m*/*z* calcd for C_20_H_18_NOS [M+H]^+^ 320.1104, found 320.1100.

*Synthesis of* ***12*:** Compound **12** was synthesized according to the synthetic method to compound **9** except that compound **7** and 2-amino-5-methylbenzenethiol were used instead of compound **4** and 2-amino-5-methoxybenzenethiol. Yield: 50%. ^1^H NMR (400 MHz, CDCl_3_) *δ* 8.11 – 8.05 (m, 3H, Ar H), 8.01 – 7.96 (m, 1H, Ar H), 7.90 (d, *J* = 8.3 Hz, 1H, Ar H), 7.64 (s, 1H, Ar H), 7.55 – 7.47 (m, 2H, Ar H), 7.46 – 7.41 (m, 2H, Ar H), 7.25 (d, *J* = 8.4 Hz, 1H, Ar H), 7.20 (s, 1H, Ar H), 2.64 (s, 3H, CH_3_), 2.55 (s, 3H, CH_3_), 2.44 (s, 3H, CH_3_); ^13^C NMR (100 MHz, CDCl_3_) *δ* 165.8 (N=C), 151.4 (Ar C), 144.4 (Ar C), 136.7 (Ar C), 134.35 (Ar C), 134.25 (Ar C), 132.1 (Ar C), 131.2 (Ar C), 131.11 (Ar C), 131.05 (Ar C), 129.4 (Ar C), 128.0, (Ar C) 127.5 (Ar C), 126.9 (Ar C), 126.1 (Ar C), 125.0 (Ar C), 124.5 (Ar C), 124.1 (Ar C), 123.6 (Ar C), 121.7 (Ar C), 120.4 (Ar C), 20.6 (CH_3_), 18.3 (CH_3_), 15.2 (CH_3_); HRMS-ESI *m*/*z* calcd for C_26_H_22_NS [M+H]^+^ 380.1467, found 380.1460.

*Synthesis of* ***13*:** Compound **13** was synthesized according to the synthetic method to compound **9** except that compound **7** was used instead of compound **4**. Yield: 10%. ^1^H NMR (400 MHz, CDCl_3_) *δ* 8.09 – 8.04 (m, 3H, Ar H), 7.99 (d, *J* = 7.4 Hz, 1H, Ar H), 7.91 (d, *J* = 8.9 Hz, 1H, Ar H), 7.55 – 7.47 (m, 2H, Ar H), 7.46 – 7.40 (m, 2H, Ar H), 7.31 (d, *J* = 2.5 Hz, 1H, Ar H), 7.21 (s, 1H, Ar H), 7.04 (d, *J* = 6.4 Hz, 1H, Ar H), 3.84 (s, 3H, OCH_3_), 2.64 (s, 3H, CH_3_), 2.55 (s, 3H, CH_3_); ^13^C NMR (100 MHz, CDCl_3_) *δ* 164.4 (N=C), 156.8 (Ar C), 147.8 (Ar C), 144.2 (Ar C), 136.7 (Ar C), 135.4 (Ar C), 132.1 (Ar C), 131.2 (Ar C), 131.1 (Ar C), 131.0 (Ar C), 129.4 (Ar C), 128.0 (Ar C), 127.6 (Ar C), 125.9 (Ar C), 125.0 (Ar C), 124.5 (Ar C), 124.1 (Ar C), 123.6 (Ar C), 122.7 (Ar C), 114.6 (Ar C), 103.2 (Ar C), 54.8 (OCH_3_), 18.3 (CH_3_), 15.2 (CH_3_); HRMS-ESI *m*/*z* calcd for C_26_H_22_NOS [M+H]^+^ 396.1417, found 396.1408.

# 3. Temporal Evolution of Endoperoxides

**Figure S1.** Half-life calculation of **ENDO-2**: 1.4 hours (at 37 ℃ in CDCl_3_)


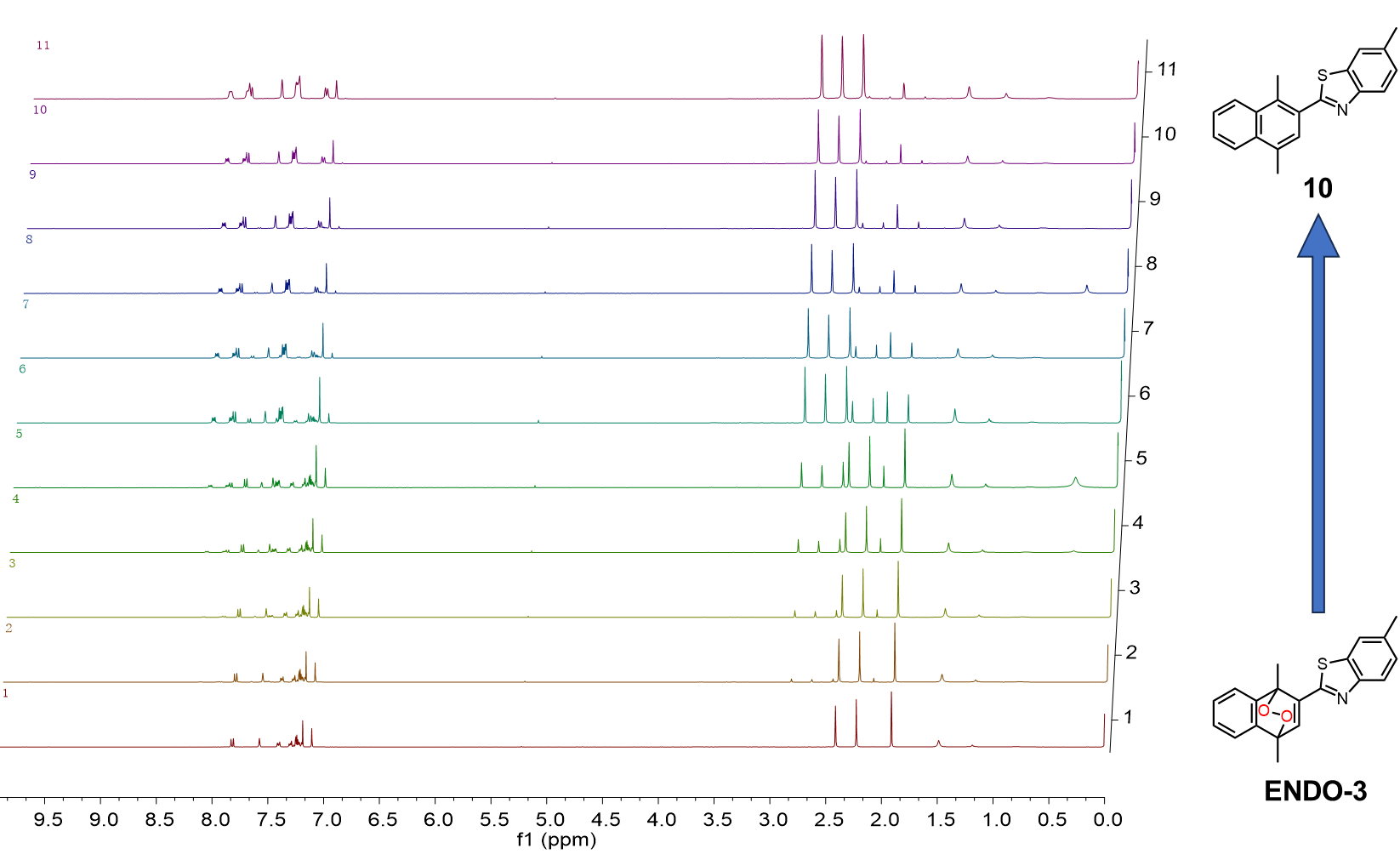


**Figure S2.** Temporal evolution of endoperoxide **ENDO-3** at 37 °C. (from bottom to top, 1: 0 hours, 2: 2 hours, 3: 4 hours, 4: 7 hours, 5: 11 hours, 6: 24 hours, 7: 36 hours, 8: 49 hours, 9: 72 hours, 10: 84 hours, 11: compound **10**).

**Figure S3.** Half-life calculation of **ENDO-3**: 18.4 hours (at 37 ℃ in CDCl_3_)


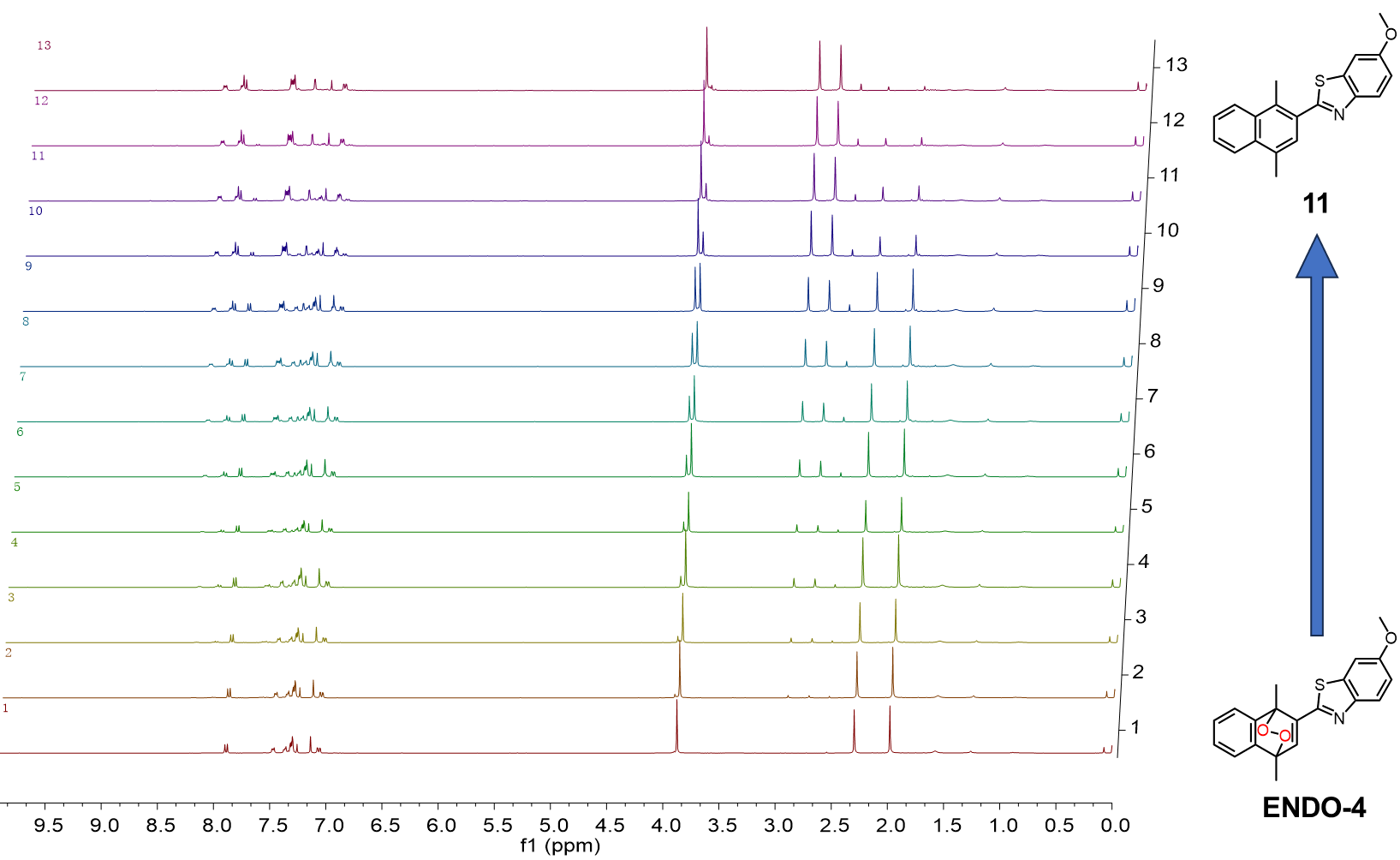


**Figure S4** Temporal evolution of endoperoxide **ENDO-4** at 37 °C. (from bottom to top, 1: 0 hours, 2: 1 hours, 3: 2 hours, 4: 3 hours, 5: 4 hours, 6: 6 hours, 7: 8 hours, 8: 10 hours, 9: 12 hours, 10: 24 hours, 11: 36 hours, 12: 48 hours, 13: compound **11**).

**Figure S5** Half-life calculation of **ENDO-4**: 15.8 hours (at 37 ℃ in CDCl_3_)


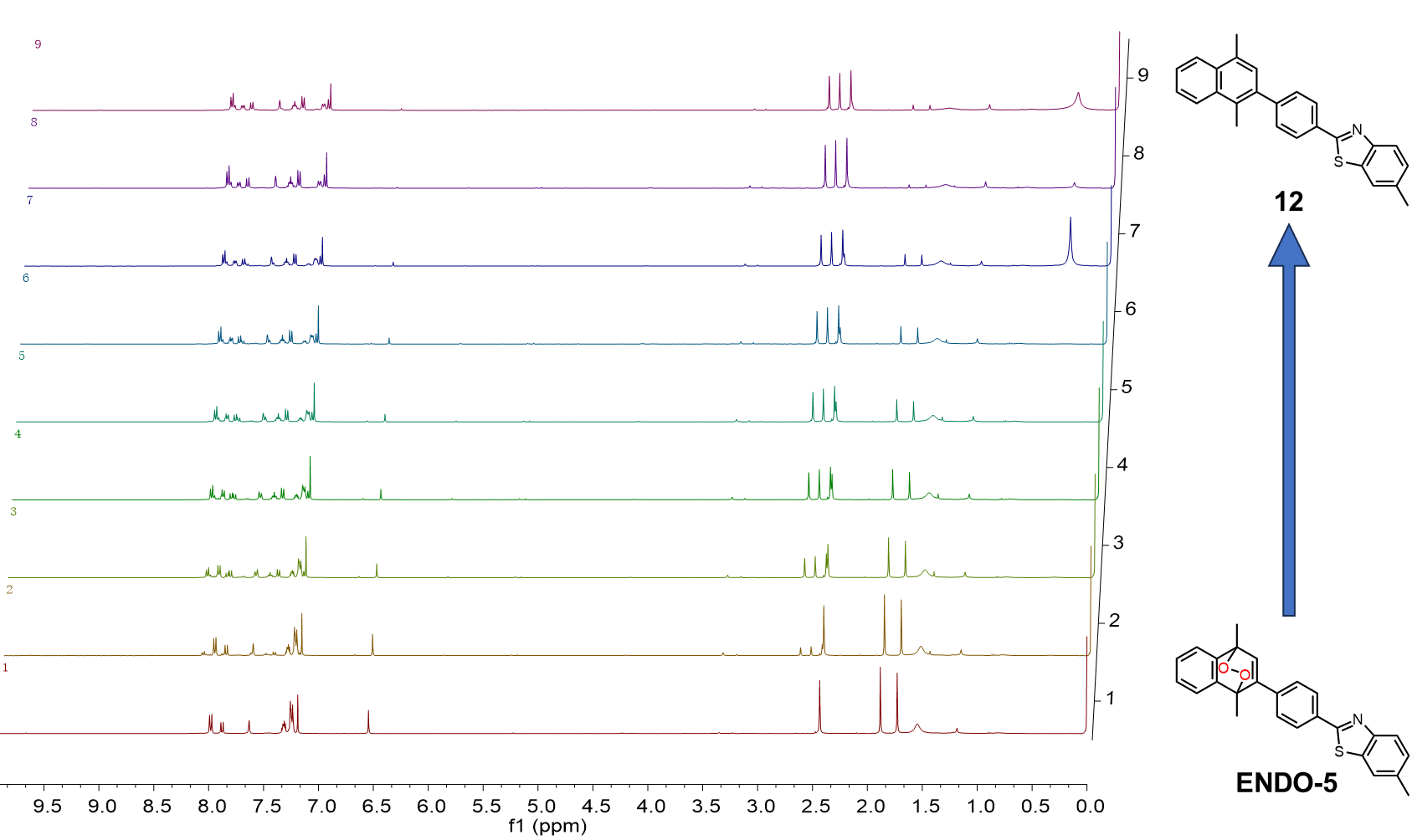


**Figure S6** Temporal evolution of endoperoxide **ENDO-5** at 37 °C. (from bottom to top, 1: 0 hours, 2: 1 hours, 3: 3 hours, 4: 5 hours, 5: 7 hours, 6: 9 hours, 7: 11 hours, 8: 24 hours, 9: compound **12**).

**Figure S7** Half-life calculation of **ENDO-5**: 6.1 hours (at 37 ℃ in CDCl_3_)


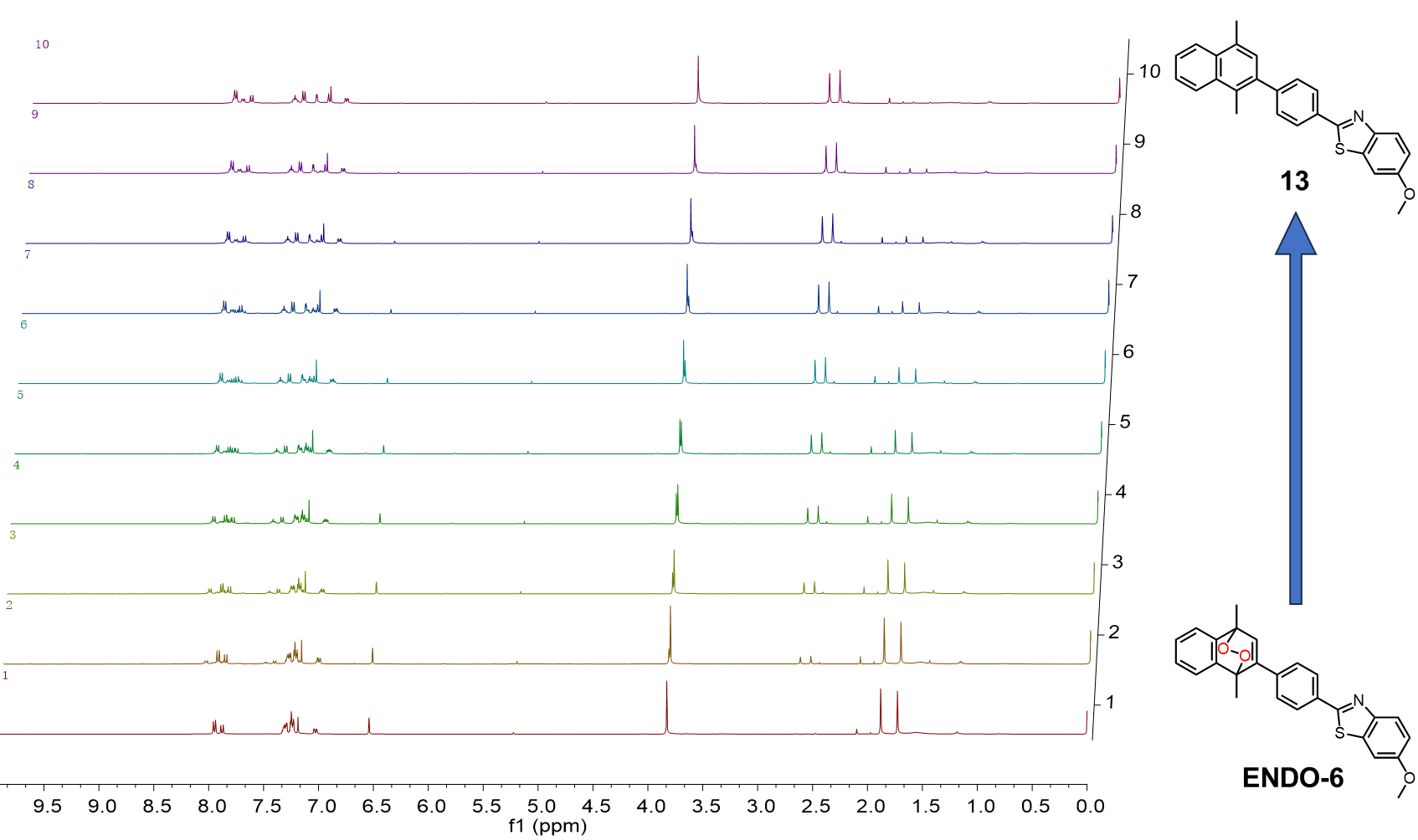


**Figure S8** Temporal evolution of endoperoxide **ENDO-6** at 37 °C. (from bottom to top, 1: 0 hours, 2: 1 hours, 3: 2 hours, 4: 3 hours, 5: 4 hours, 6: 6 hours, 7: 8 hours, 8: 10 hours, 9: 12 hours, 10: compound **13**).

**Figure S9** Half-life calculation of **ENDO-6**: 3.8 hours (at 37 ℃ in CDCl_3_).

# 4. Detection of Singlet Oxygen

**Figure S10** Time dependent UV-Vis spectra of DPBF in the presence of 500 µM **ENDO-3** in DMF at 37 ℃.

**Figure S11** Time dependent UV-Vis spectra of DPBF in the presence of 500 µM **ENDO-4** in DMF at 37 ℃.

**Figure S12** Time dependent UV-Vis spectra of DPBF in the presence of 500 µM **ENDO-5** in DMF at 37 ℃.

**Figure S13** Time dependent UV-Vis spectra of DPBF in the presence of 500 µM **ENDO-6** in DMF at 37 ℃.

# 5. Dynamic Light Scattering (DLS) Measurement


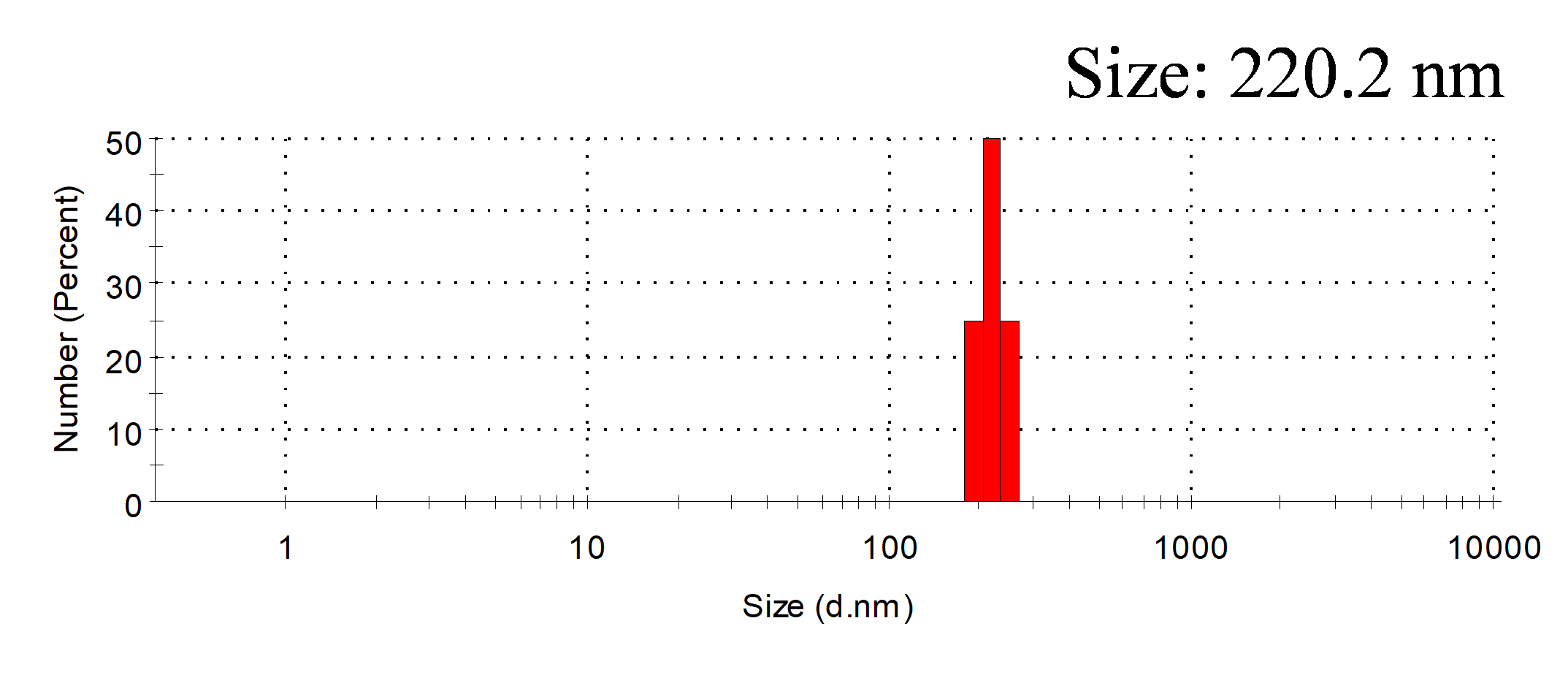


**Figure S14** DLS size distribution of monomeric Aβ_1–42_.


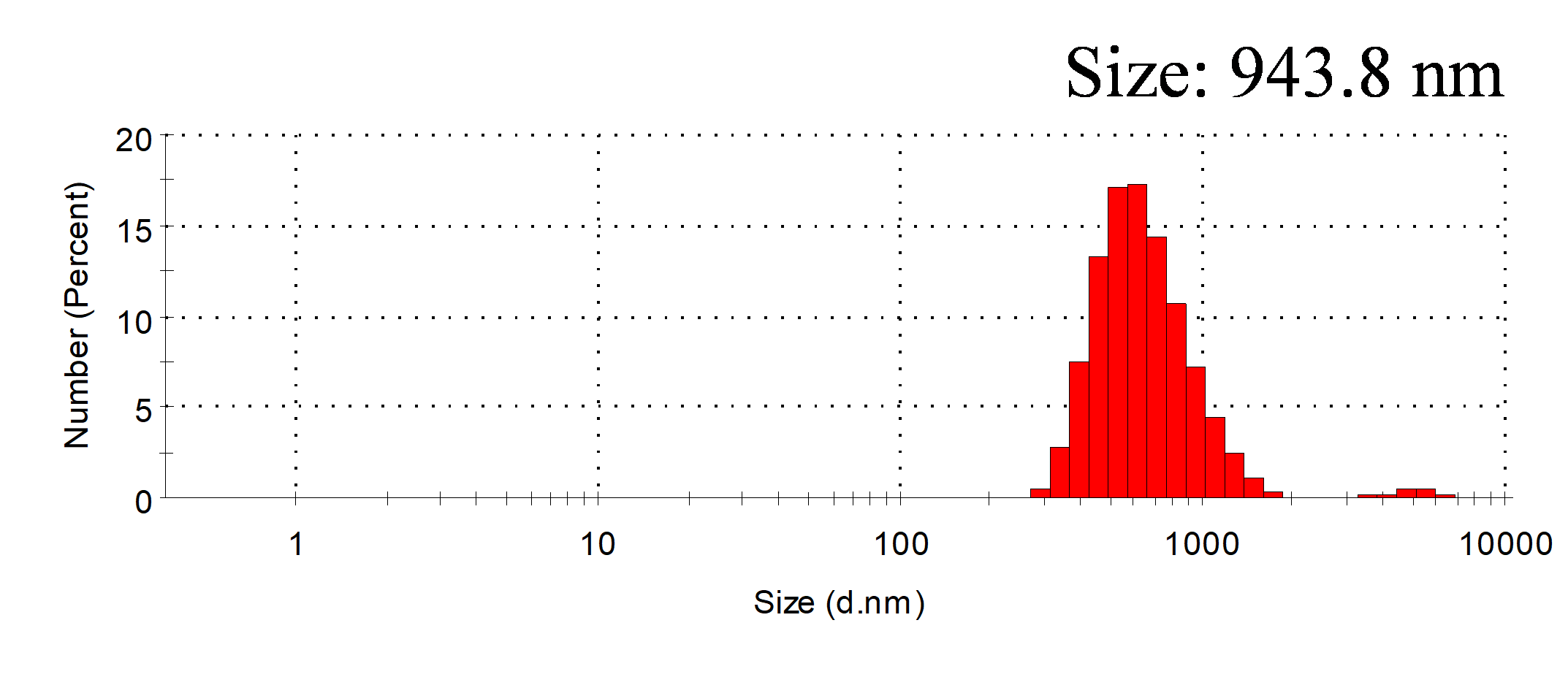


**Figure S15** DLS size distribution of Aβ_1–42_ incubated at 37 ℃ for 48 h.


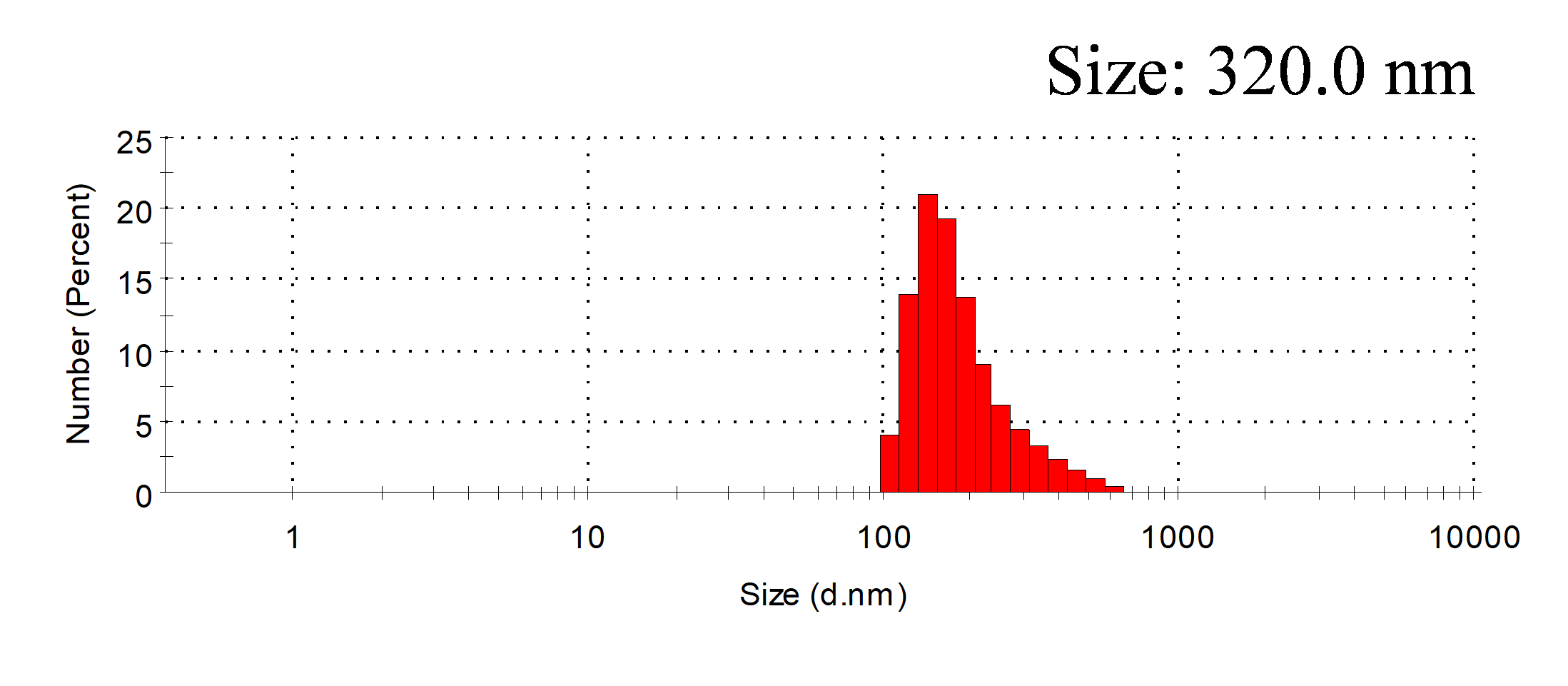


**Figure S16** DLS size distribution of Aβ_1–42_ incubated with **ENDO-2** at 37 ℃ for 48 h.


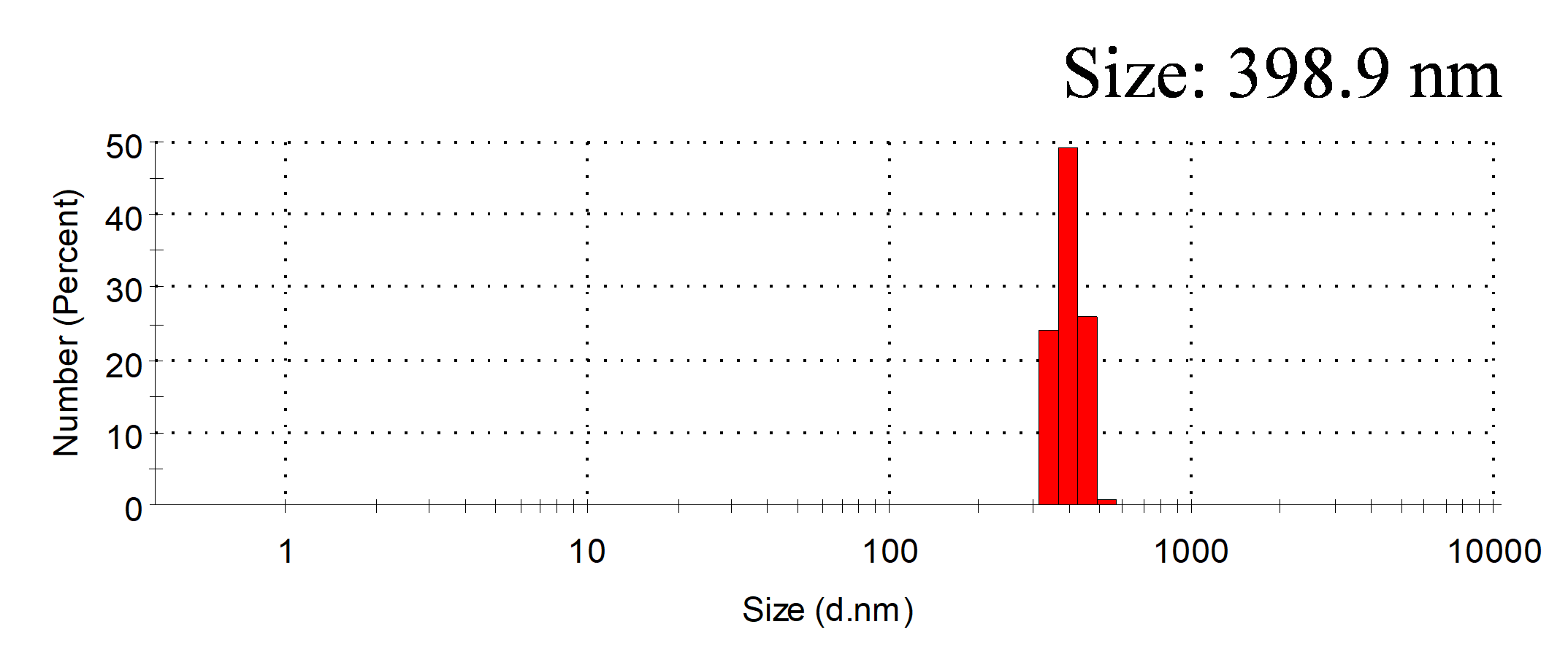


**Figure S17** DLS size distribution of Aβ_1–42_ incubated with **ENDO-3** at 37 ℃ for 48 h.


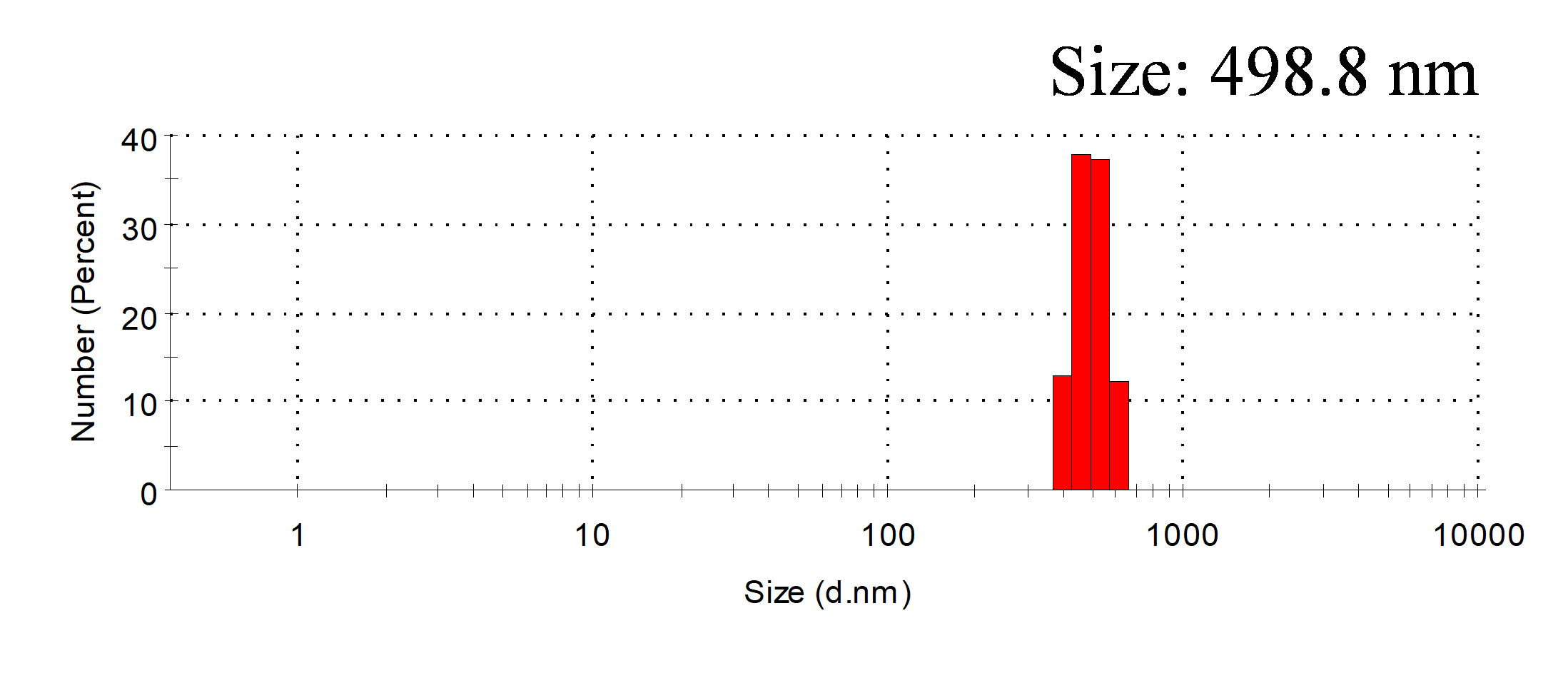


**Figure S18** DLS size distribution of Aβ_1–42_ incubated with **ENDO-4** at 37 ℃ for 48 h.


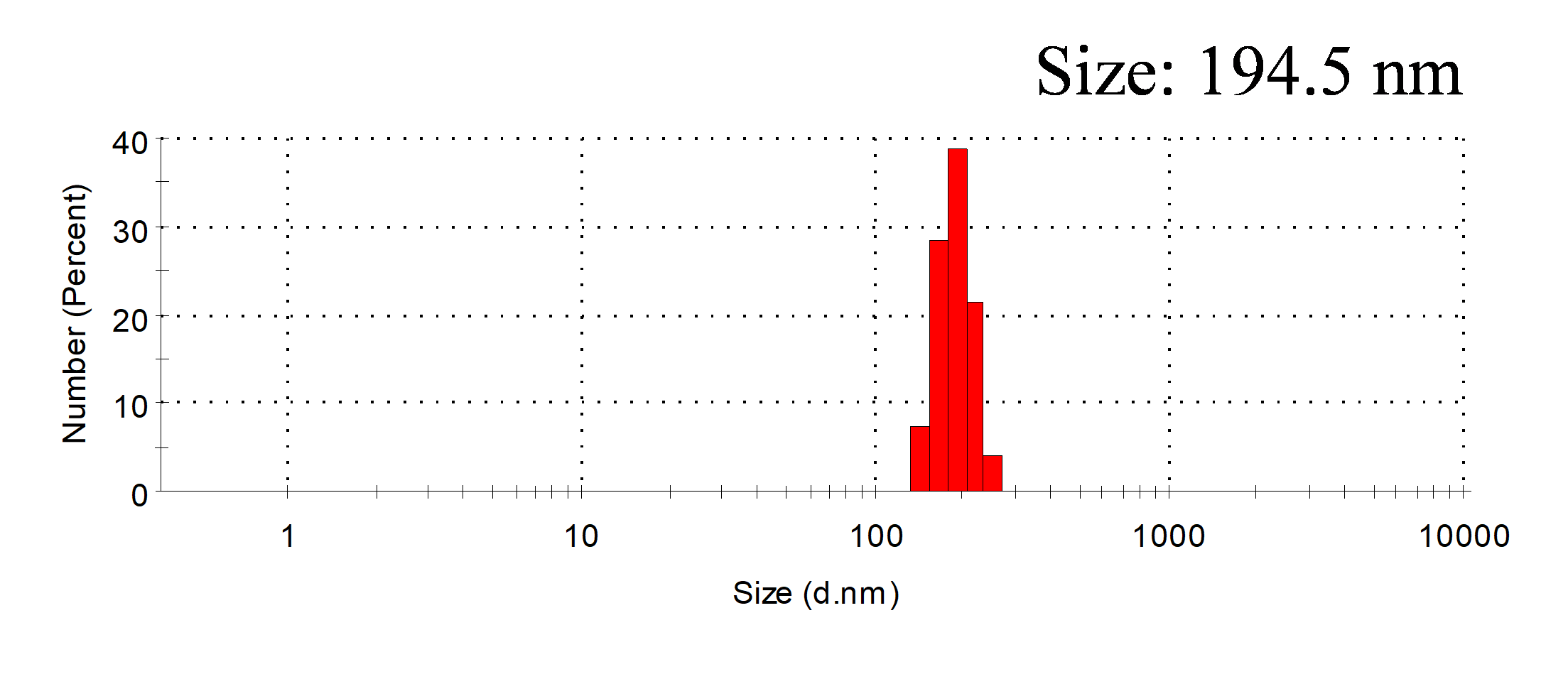


**Figure S19** DLS size distribution of Aβ_1–42_ incubated with **ENDO-5** at 37 ℃ for 48 h.


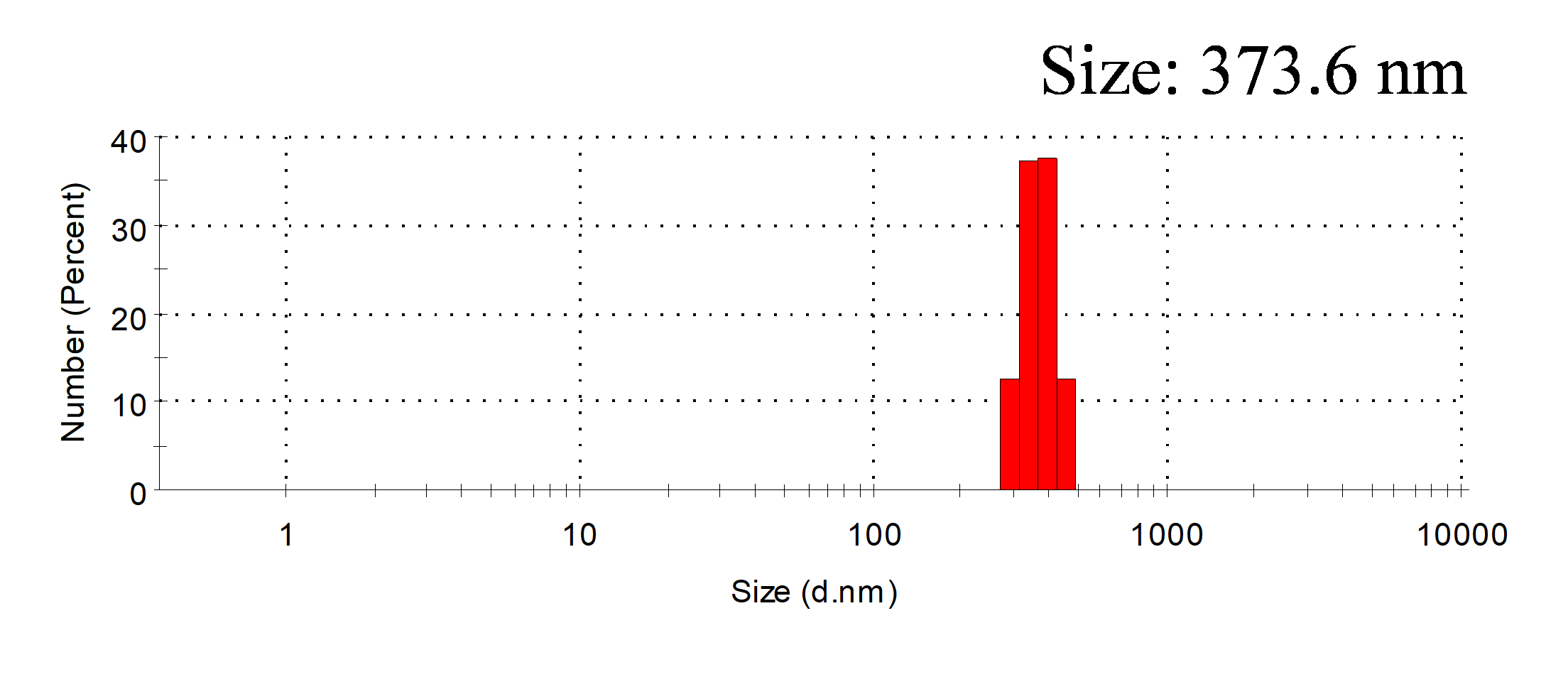


**Figure S20** DLS size distribution of Aβ_1–42_ incubated with **ENDO-6** at 37 ℃ for 48 h.

# 6. Cytotoxicity of Endoperoxides

PC12 cells were seeded in 96-well plate (5000 cells per well) and incubated at 37 ℃ for 24 h in DMEM medium. And then, the cell was co-incubated with various concentration of compounds **8**, **9**, **10**, **11**, **12** and **13**, or endoperoxides **ENDO-1**, **2**, **3**, **4**, **5** and **6** for another 24 h at 37 ℃, respectively. 3′-(4,5-dimethylthiazol-2-yl)-2,5-diphenyltetrazoliumbromide (MTT) in PBS (5 mg/mL, 20 μL) were added. With further incubation at 37 °C for 4 h, the medium was removed. DMSO (150 μL) was added to each well and the absorbance at 570 nm was measured with a microplate reader (SpectraMax i3x, Molecular Devices).

**Figure S21** Cell viability of PC12 cells after incubation with various concentrations of compound **8**, **9**, **10**, **11**, **12** and **13**.

**Figure S22** Cell viability of PC12 cells after incubation with various concentrations of **ENDO-1**, **2**, **3**, **4**, **5** and **6**.

# 7. Reference

[1] H. Wu, Z. Liu, Y. Shao, G. Li, Y. Pan, L. Wang, E. Akkaya, *Chem. Commun.* **2022**, *58*, 3747.

[2] E. Ucar, D. Xi, O. Seven, C. Kaya, X. Peng, W. Sun, E. Akkaya, *Chem. Commun.* **2019**, *55*, 13808.

# 8. NMR Spectra


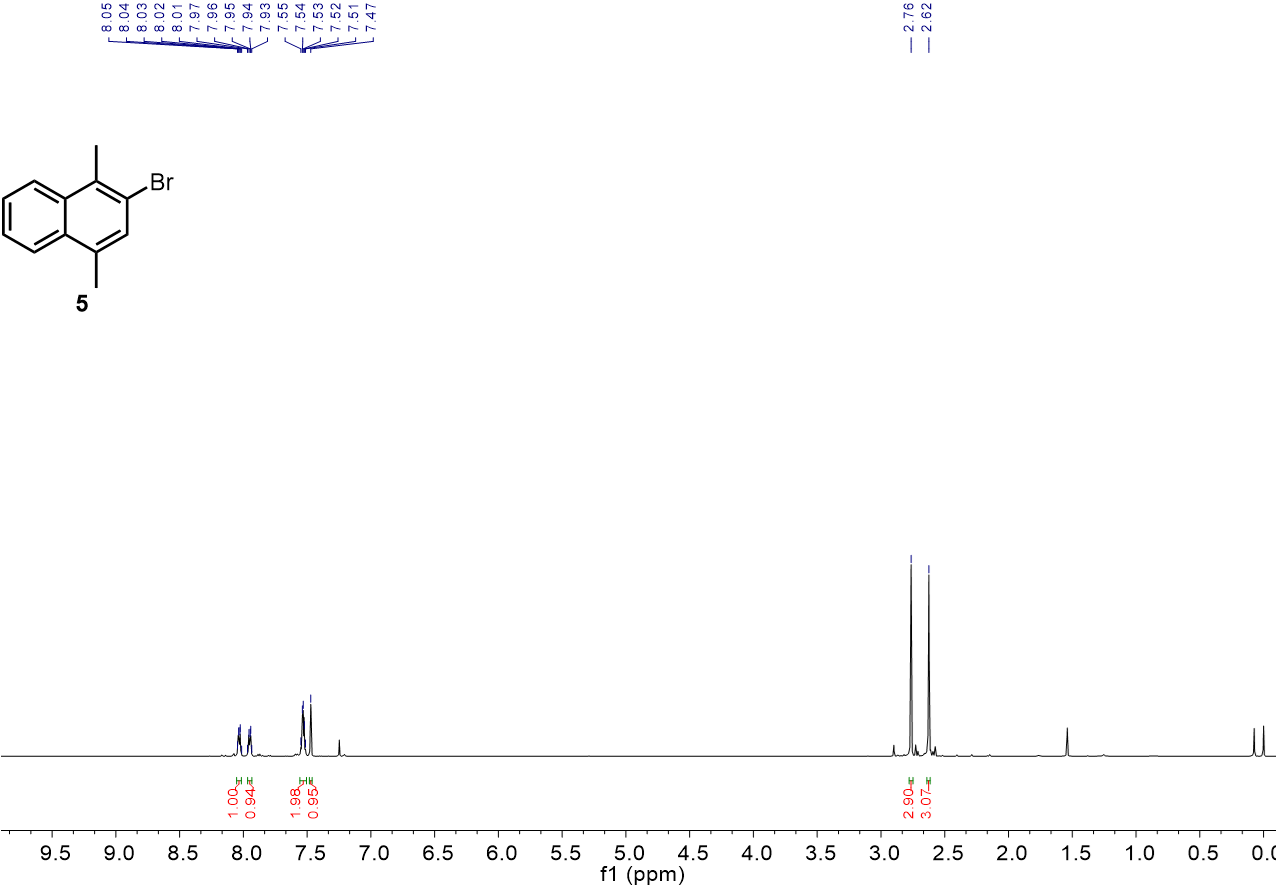


^1^H NMR spectrum of compound **5**

^1^H NMR spectrum of compound **6**

^13^C NMR spectrum of compound **6**


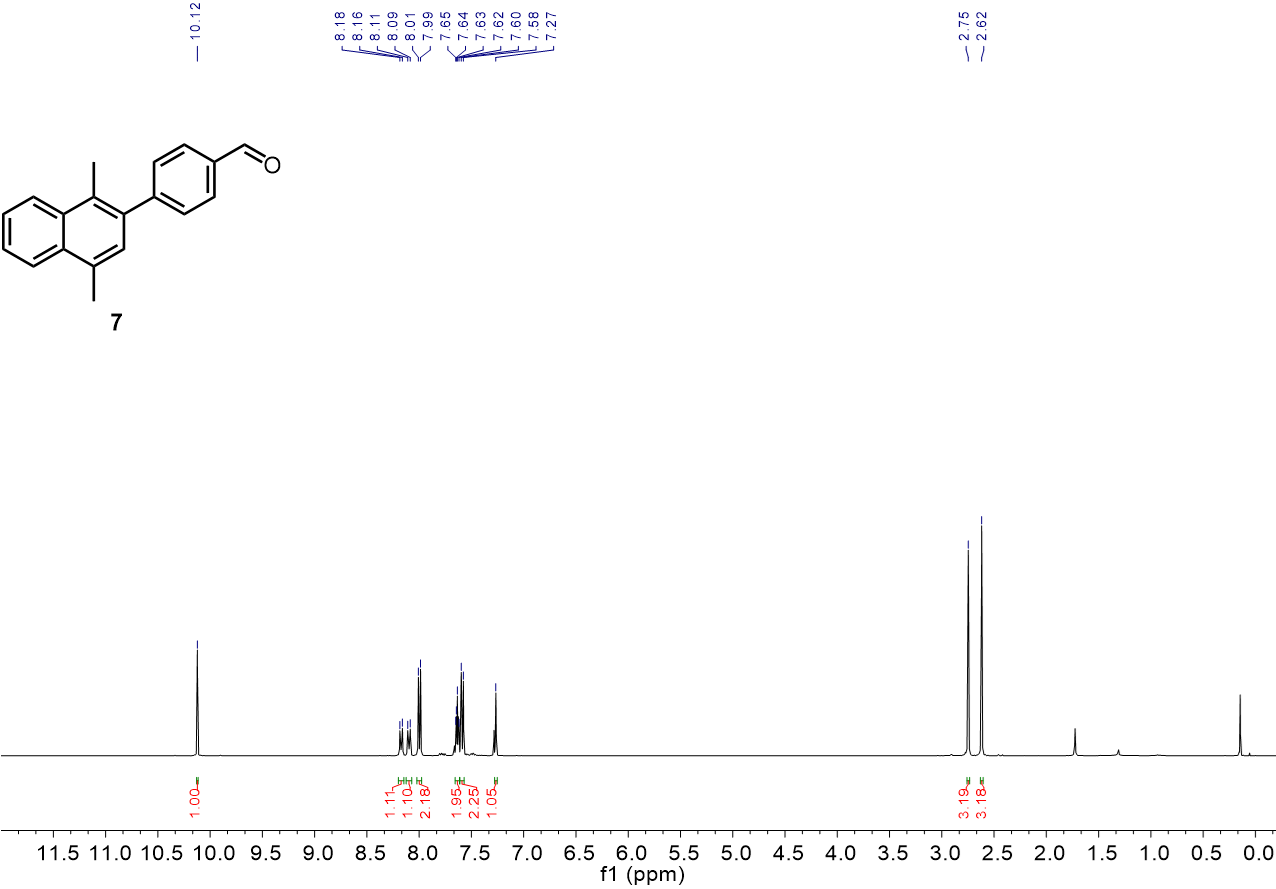


^1^H NMR spectrum of compound **7**

^13^C NMR spectrum of compound **7**

^1^H NMR spectrum of compound **9**

^13^C NMR spectrum of compound **9**


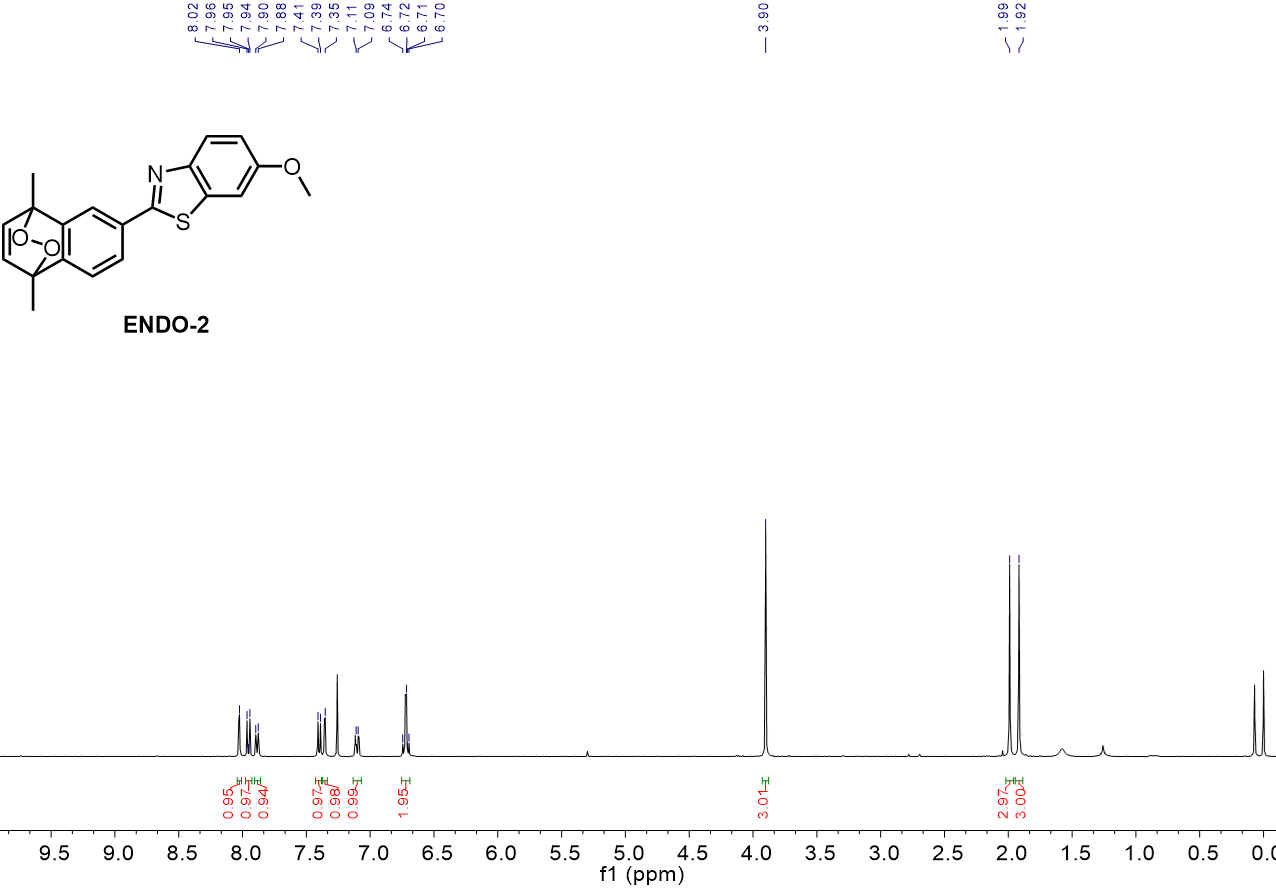


^1^H NMR spectrum of compound **ENDO-2**

^13^C NMR spectrum of compound **ENDO-2**


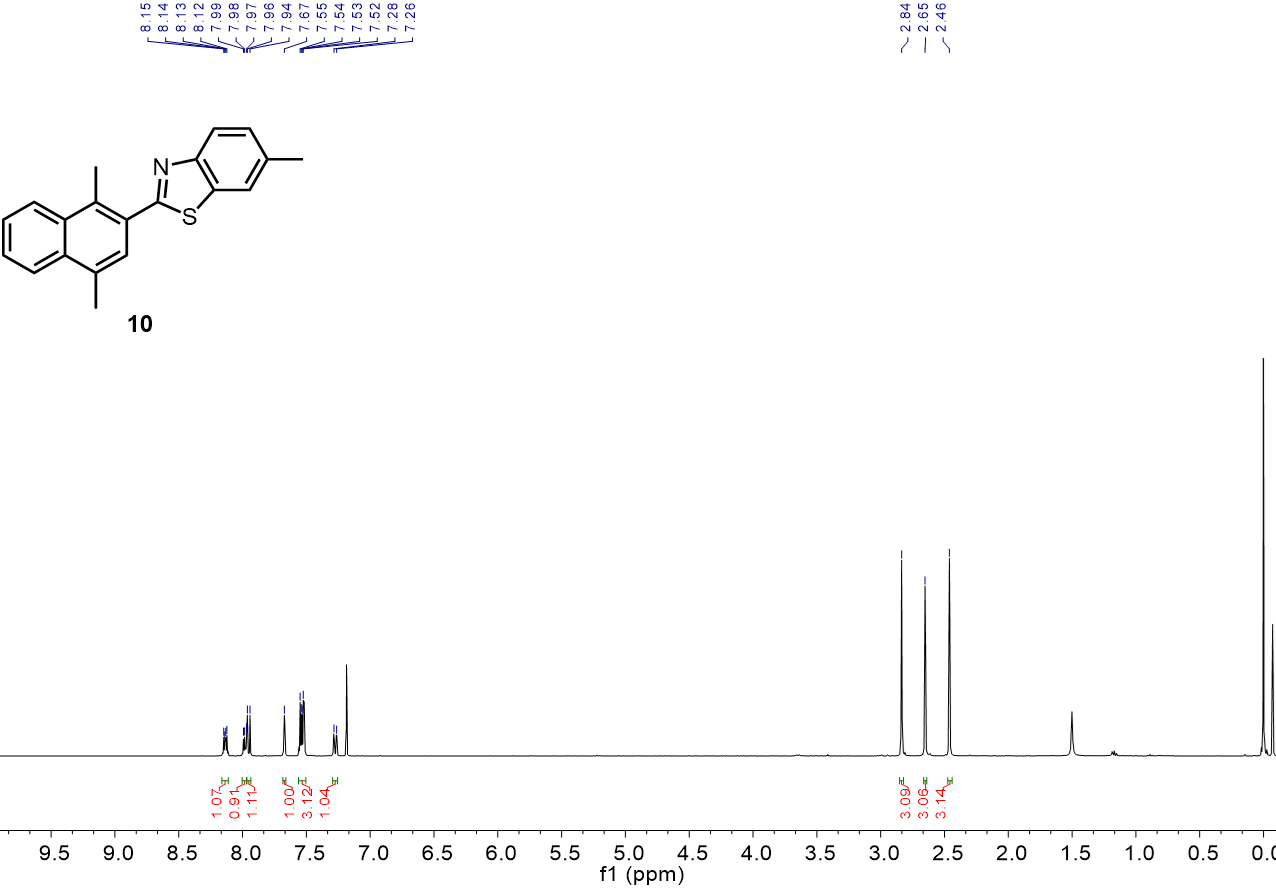


^1^H NMR spectrum of compound **10**

^13^C NMR spectrum of compound **10**


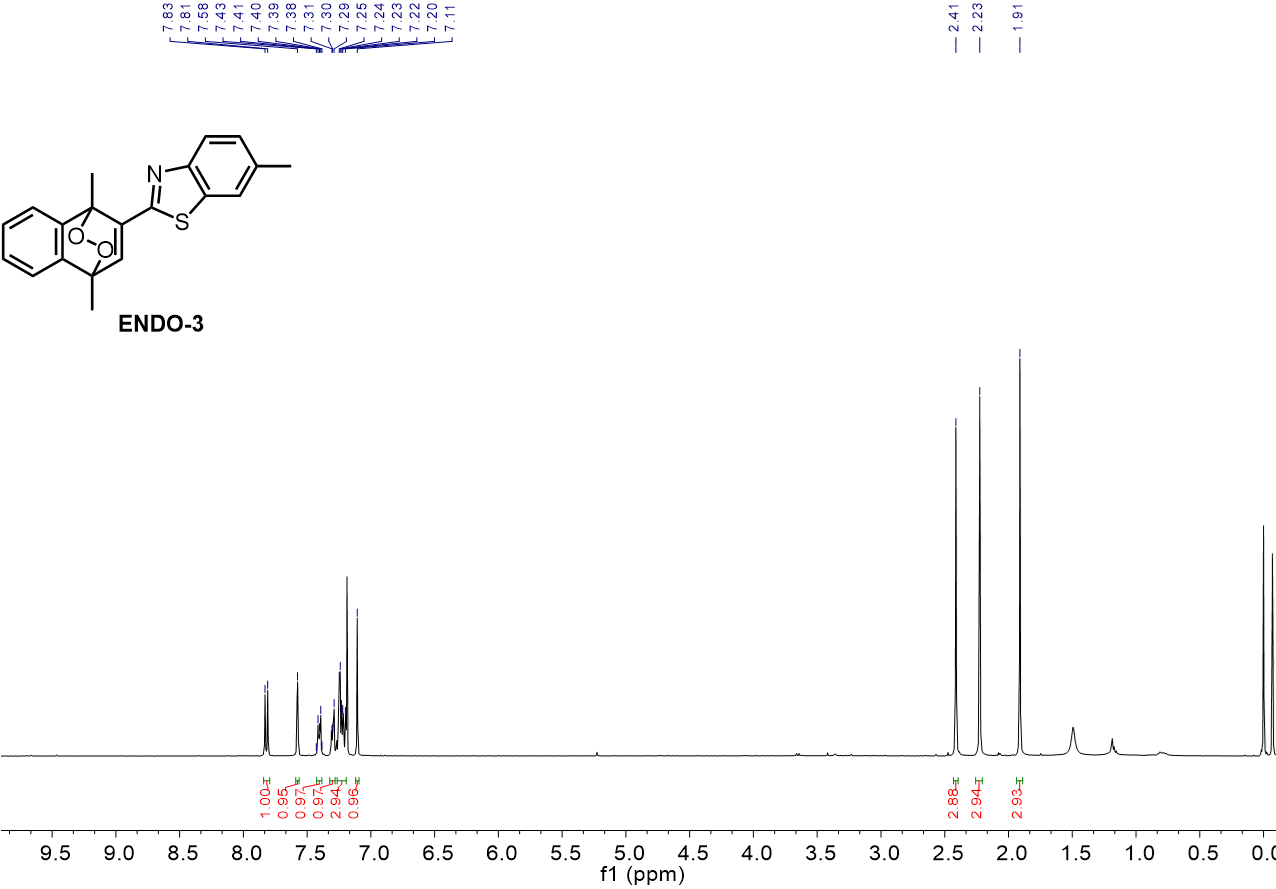


^1^H NMR spectrum of compound **ENDO-3**

^13^C NMR spectrum of compound **ENDO-3**


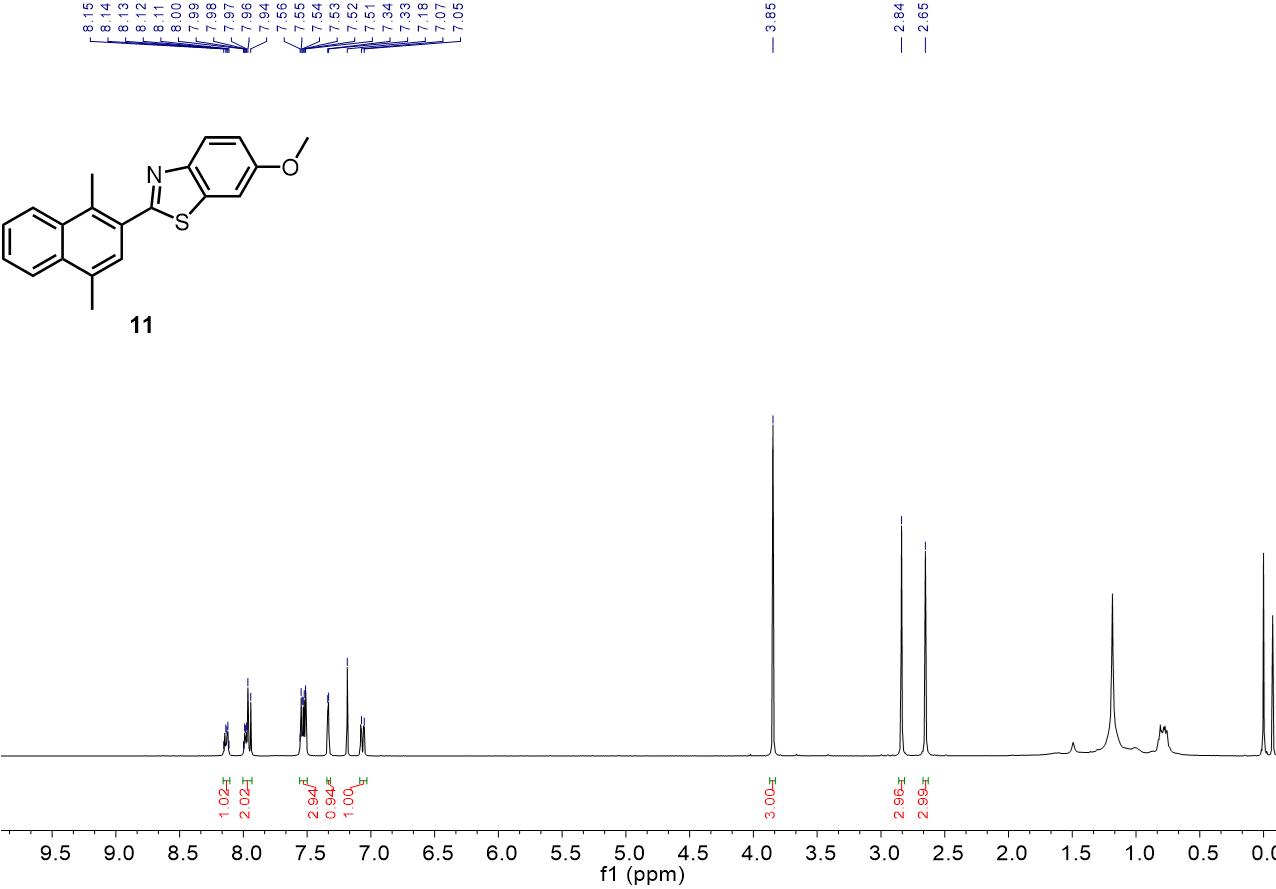


^1^H NMR spectrum of compound **11**

^13^C NMR spectrum of compound **11**


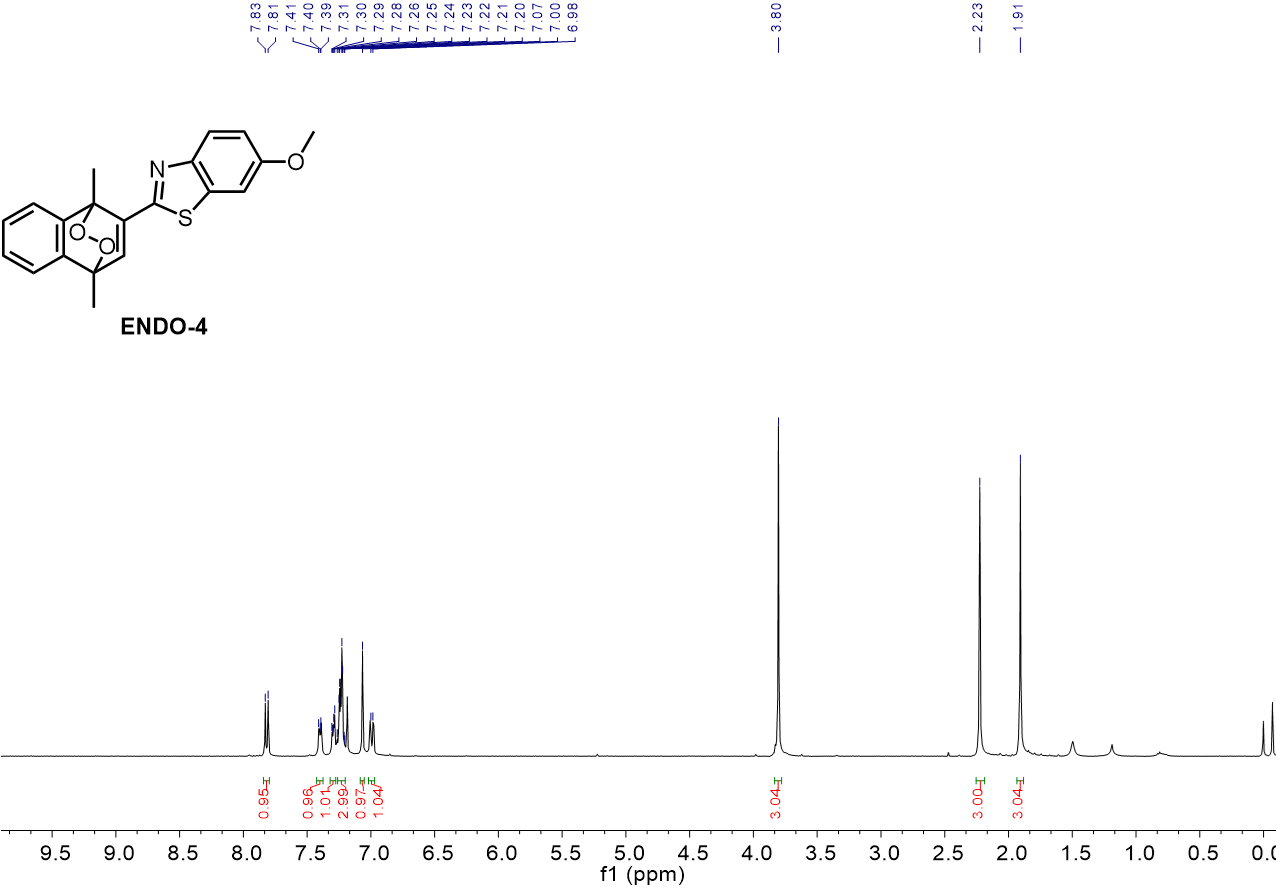


^1^H NMR spectrum of compound **ENDO-4**

^13^C NMR spectrum of compound **ENDO-4**


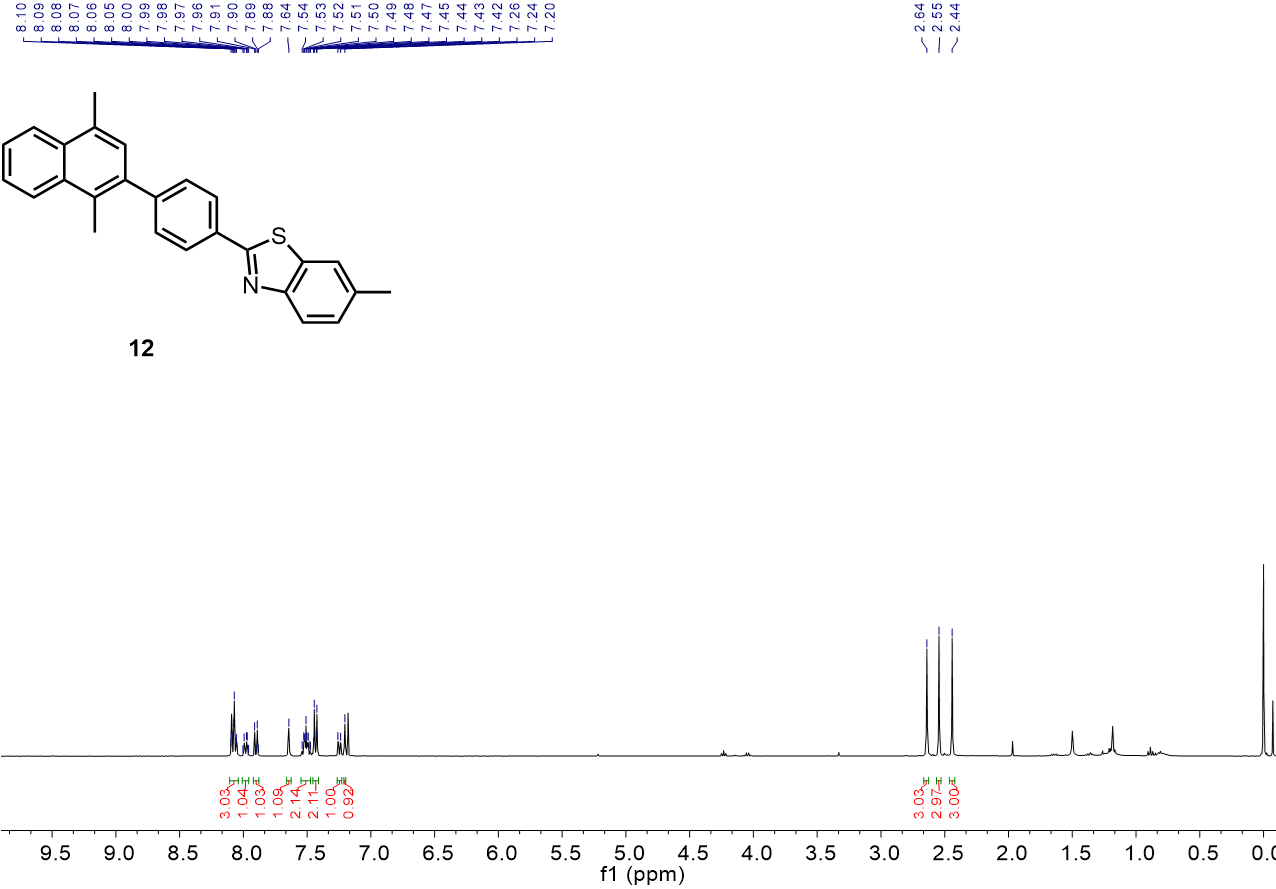


^1^H NMR spectrum of compound **12**

^13^C NMR spectrum of compound **12**


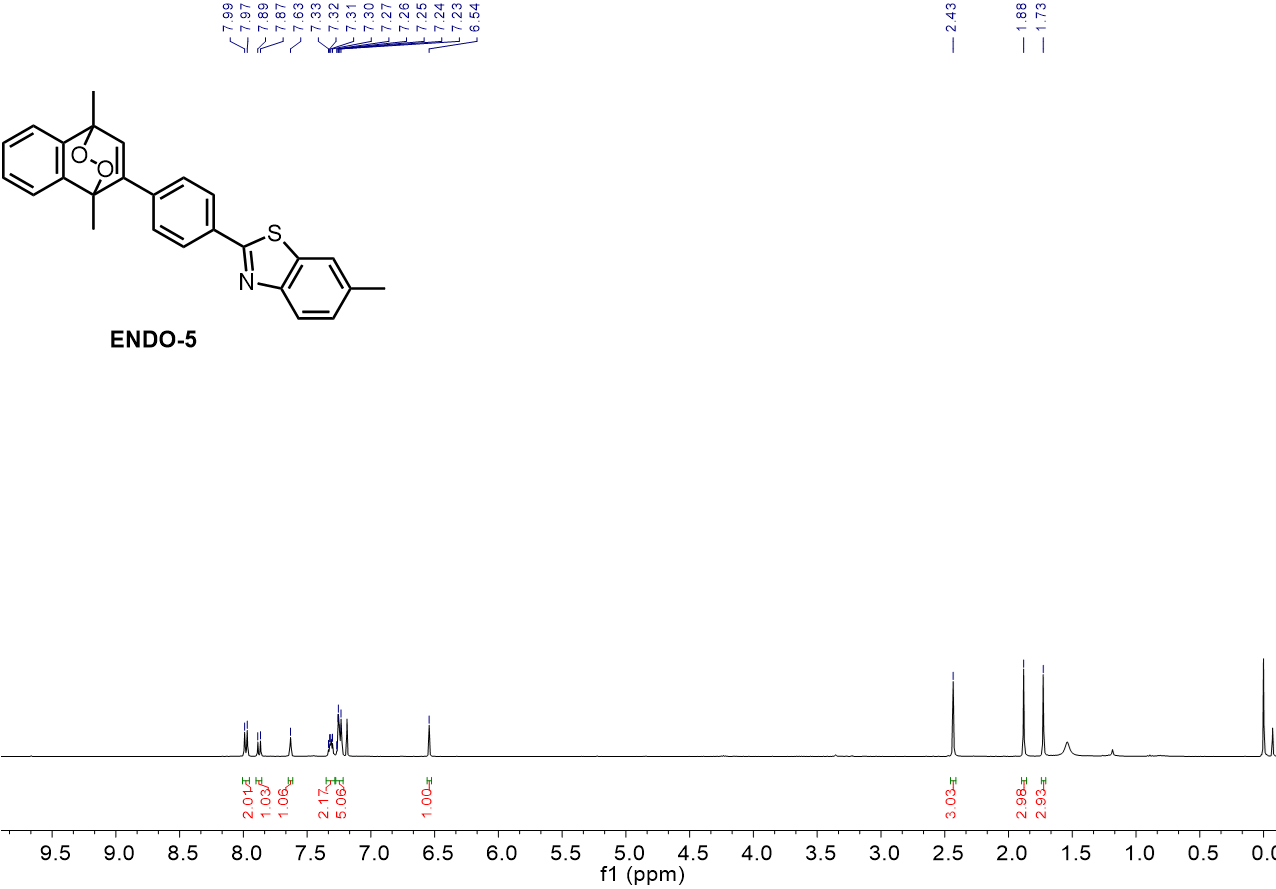


^1^H NMR spectrum of compound **ENDO-5**

^13^C NMR spectrum of compound **ENDO-5**


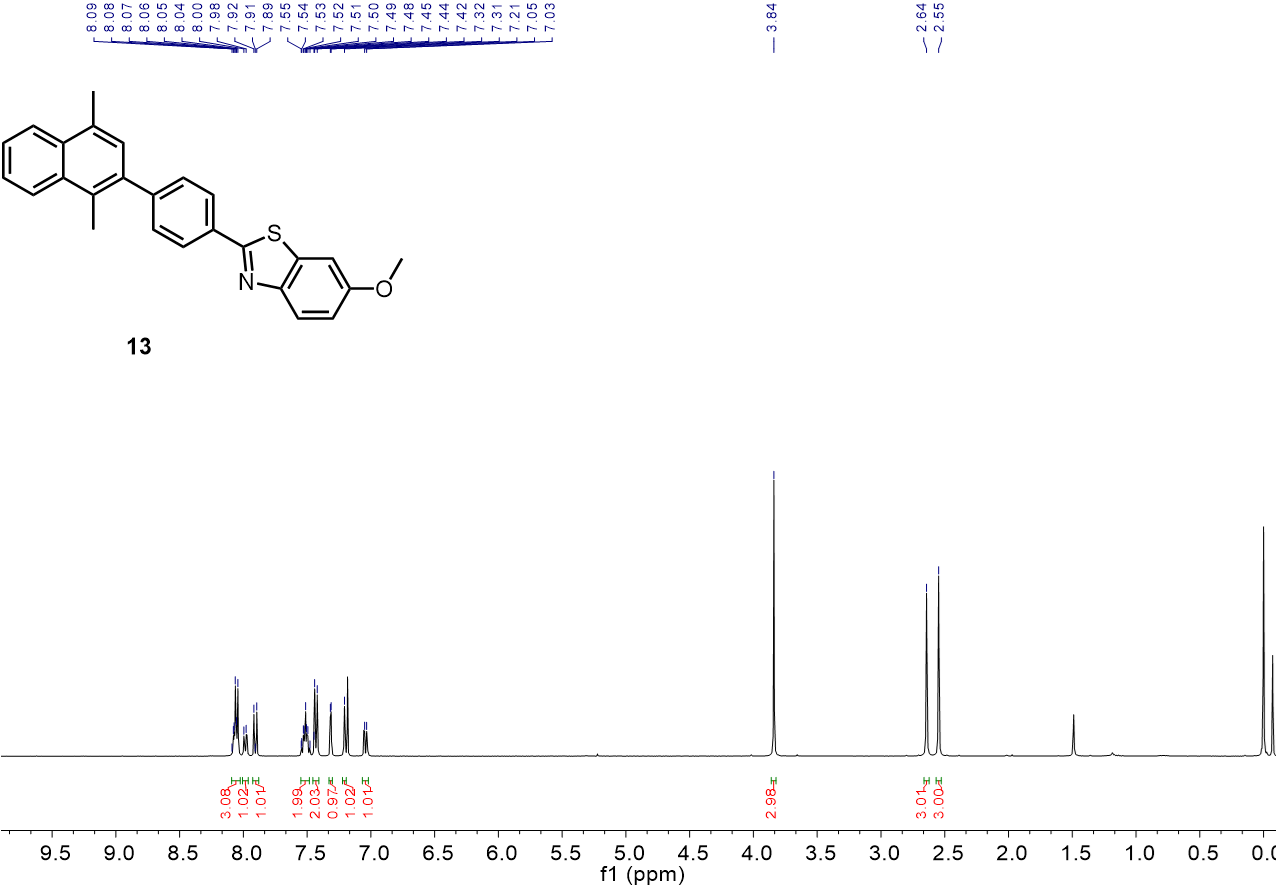


^1^H NMR spectrum of compound **13**

^13^C NMR spectrum of compound **13**


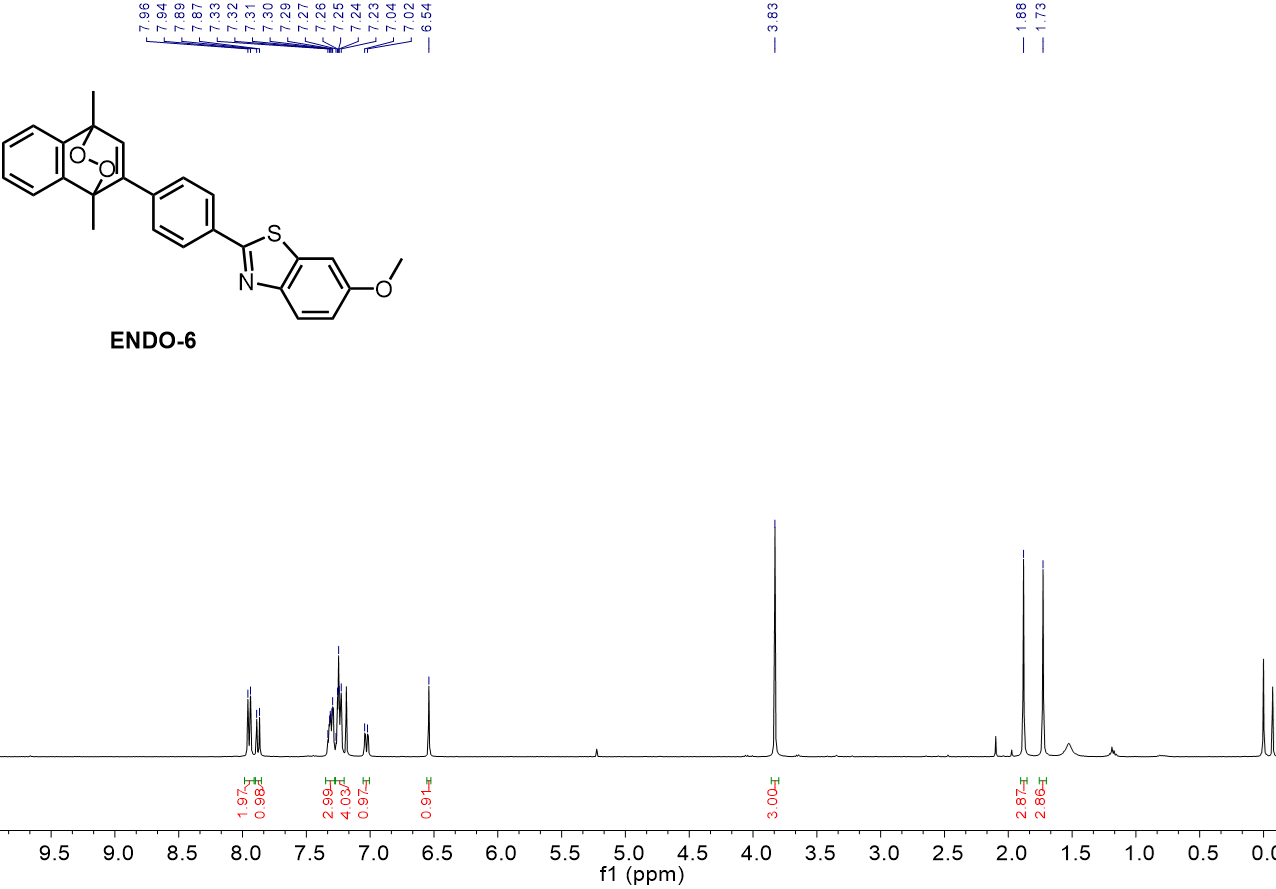


^1^H NMR spectrum of compound **ENDO-6**

^13^C NMR spectrum of compound **ENDO-6**
